# Supplementary figures and images for: The cell cortex-localized protein CHDP-1 is required for dendritic development and transport in C. elegans neurons
Source: PLoS Genet. 2022 Sep 20;18(9):e1010381. doi: 10.1371/journal.pgen.1010381 (PMC9524629; doi:10.1371/journal.pgen.1010381)

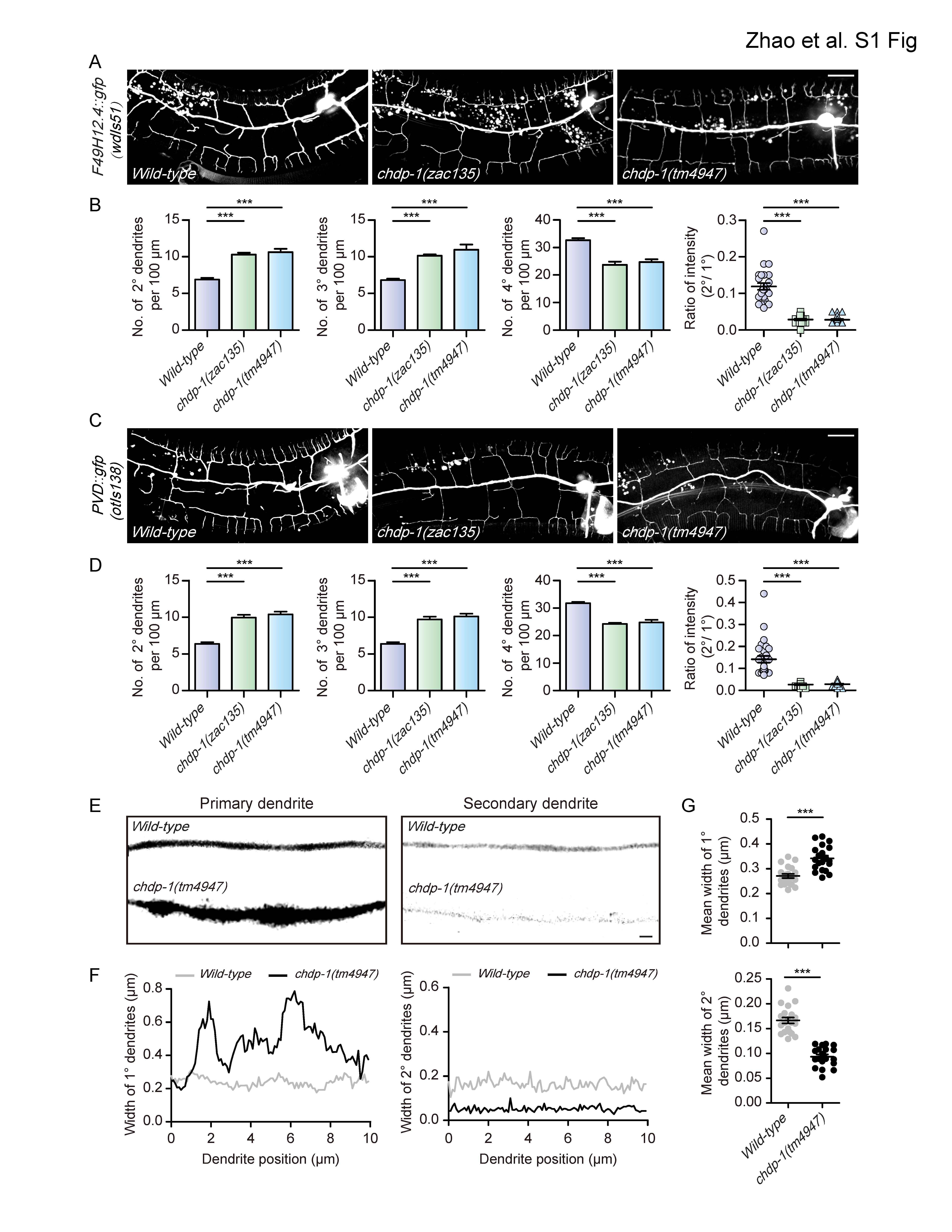

Supplement: S1 Fig — (A) Confocal images showing the PVD morphologies of wild-type (left), chdp-1(zac135) (middle) and chdp-1(tm4947) (right) animals labeled using wdIs51 which expresses cytosolic GFP driven by the F49H12.4 promoter in PVD neurons and a few other neurons. Scale bar: 20 μm. (B) Quantification of the number of 2o, 3o and 4o branches in a 100 μm area anterior to the PVD cell body, and the ratio of the intensity in the 2o branches to that of the primary dendrites in wild-type, chdp-1(zac135) and chdp-1(tm4947). The dendrites are labeled using wdIs51. Error bars, SEM. ***p < 0.001 by one-way ANOVA with the Tukey correction. n = 20–30 for each genotype. (C) Confocal images showing the PVD morphologies of wild-type (left), chdp-1(zac135) (middle) and chdp-1(tm4947) (right) animals labeled using otIs138, which expresses cytosolic GFP driven by the ser2prom3 promoter (the expression level is much lower than that of wyIs592). Scale bar: 20 μm. (D) Quantification of the number of 2o, 3o and 4o branches in a 100 μm area anterior to the PVD cell body, and the ratio of the intensity in the 2o branches to that of the primary dendrites in wild-type, chdp-1(zac135) and chdp-1(tm4947). The dendrites are labeled using otIs138. Error bars, SEM. ***p < 0.001 by one-way ANOVA with the Tukey correction. n = 20–30 for each genotype. (E) Images taken by the STED super-resolution microscopy for primary dendrites (left) and secondary dendrites (right) in wild-type and chdp-1(tm4947). Note that the orientation for the 2o branches is as following: left: close to the 1o dendrite. Right: close to the 3o branches. Scale bar: 500 nm. (F) Plots of the width along the representative primary dendrite and 2o branches in a 10 μm area. (G) Quantification of the mean width of the primary dendrite and 2o branches in wild-type and chdp-1(tm4947). Error bars: SEM. ***p < 0.001 by Student’s t test. n = 20 for each genotype. (TIFF) [file pgen.1010381.s001.tiff]

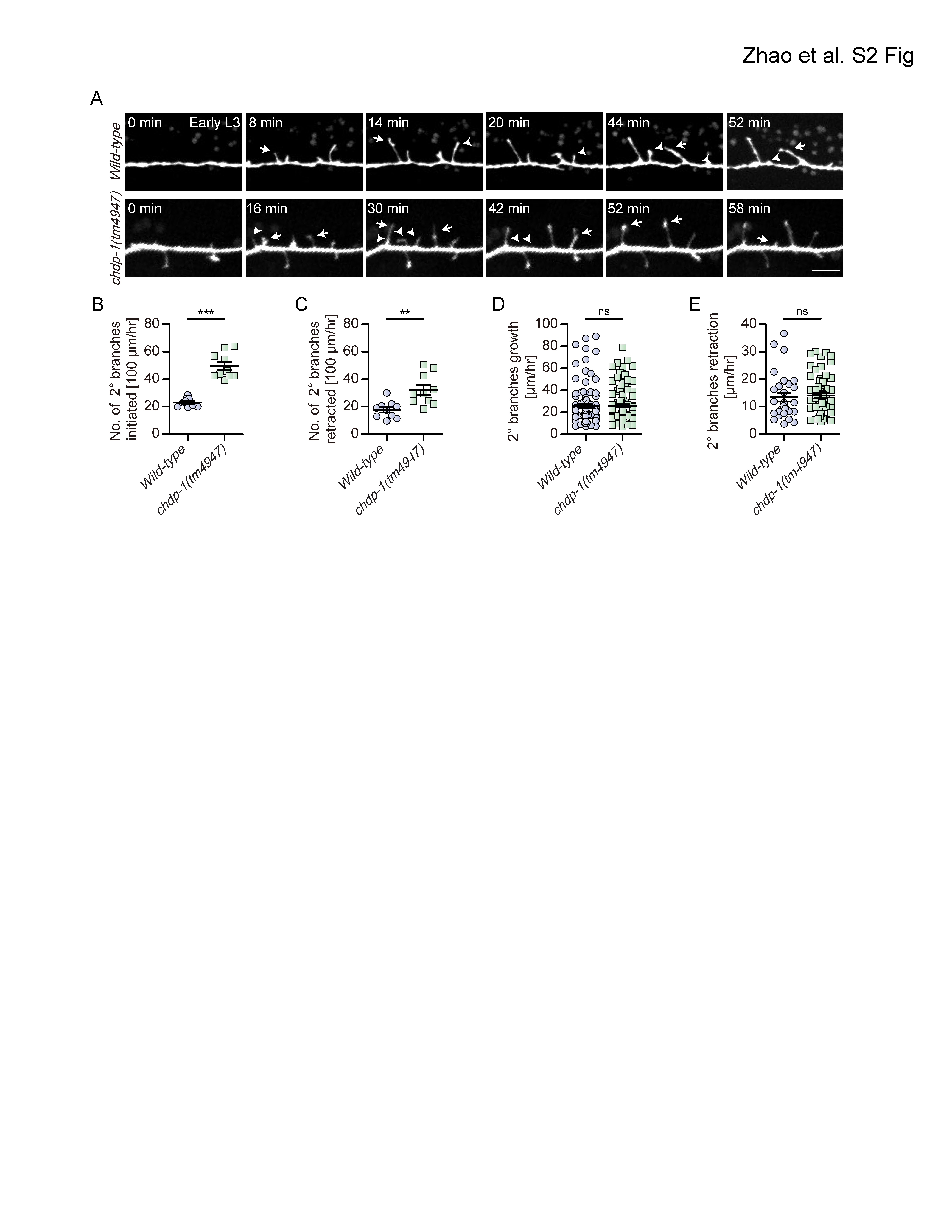

Supplement: S2 Fig — (A) Confocal images from time-lapse movies showing dendrite branching, outgrowth and retraction in wild-type (upper) and chdp-1(tm4947) mutant animals (lower) during early L3 stage. Arrows: 2o outgrowth, arrowheads: 2o retraction. Scale bar: 5 μm. (B-C) Quantification of the number of 2o branches initiation (B) and retraction (C) per hour in a 100 μm area anterior to the PVD cell body. Error bars: SEM. ***p < 0.001 by Student’s t-test. n = 10 animals for each genotype. (D-E) Quantification of the speed of 2o dendrite growth (D) and retraction (E). Error bars: SEM. ns: non-significant by Student’s t-test. For D, n = 100 branches for each genotype. For E, n = 36 branches for wild-type, and n = 29 branches for chdp-1(tm4947). (TIF) [file pgen.1010381.s002.tif]

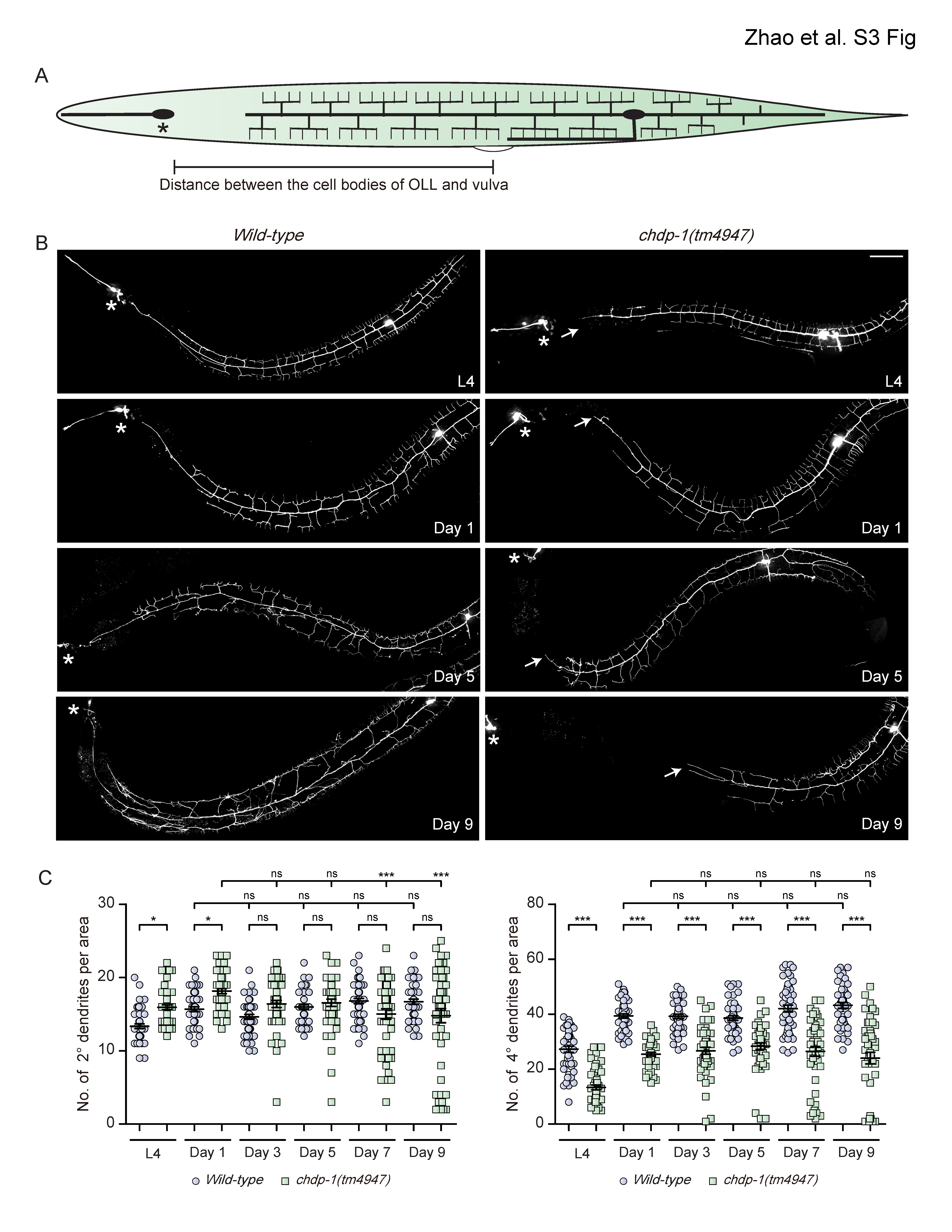

Supplement: S3 Fig — (A) A cartoon showing the simplified morphologies and locations of OLL neurons in the head region and PVD neurons. Area is defined to the distance between the cell bodies of OLL and vulva. (B) Confocal images of the PVD dendrites in wild-type and chdp-1(tm4947) mutants at the fourth larval, 1 day-old adult (DOA), 5 DOA, 9 DOA stages. Arrows: the distal tip of the anterior primary dendrite. Asterisks: cell bodies of OLL neurons. Scale bar: 50 μm. (C) Quantification of the number of 2o and 4o branches per area in wild-type and chdp-1(tm4947) mutant at six different developmental stages, including L4, 1 DOA, 3 DOA, 5 DOA, 7 DOA and 9 DOA. Error bars: SEM. *p < 0.05, ***p < 0.001 by one-way ANOVA with the Tukey correction. ns: non-significant. n = 50–60 animals for each group. (TIF) [file pgen.1010381.s003.tif]

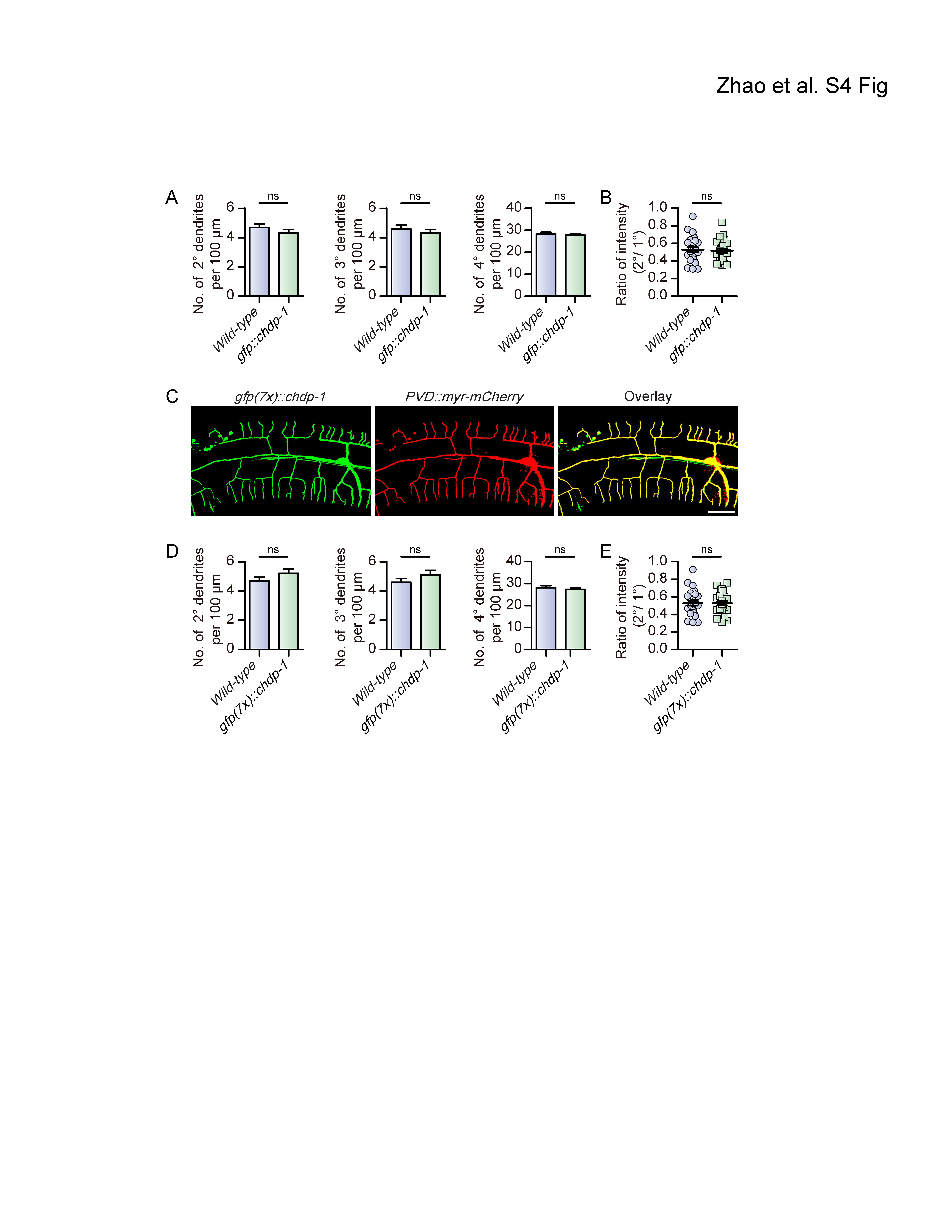

Supplement: S4 Fig — (A-B) Quantification of the number of (A) 2o, 3o and 4o branches in a 100 μm area anterior to the PVD cell body, and (B) the ratio of the intensity in the 2o branches to that of the primary dendrites in wild-type and gfp::chdp-1 knock-in animals. Error bars: SEM. ns: non-significant by Student’s t-test. n = 20–30 for each genotype. (C) Confocal images showing the expression patterns of GFP(7x)::CHDP-1 (left), mCherry (middle) and overlay (right) in the PVD neurons. Scale bar: 20 μm. (D-E) Quantification of (D) the number of 2o, 3o and 4o branches in a 100 μm area anterior to the PVD cell body, and (E) the ratio of the intensity of the 2° branches to that of the primary dendrite in wild-type and gfp(7x)::chdp-1 knock-in animals. Error bars: SEM. ns: non-significant by Student’s t-test. n = 20–30 for each genotype. (TIF) [file pgen.1010381.s004.tif]

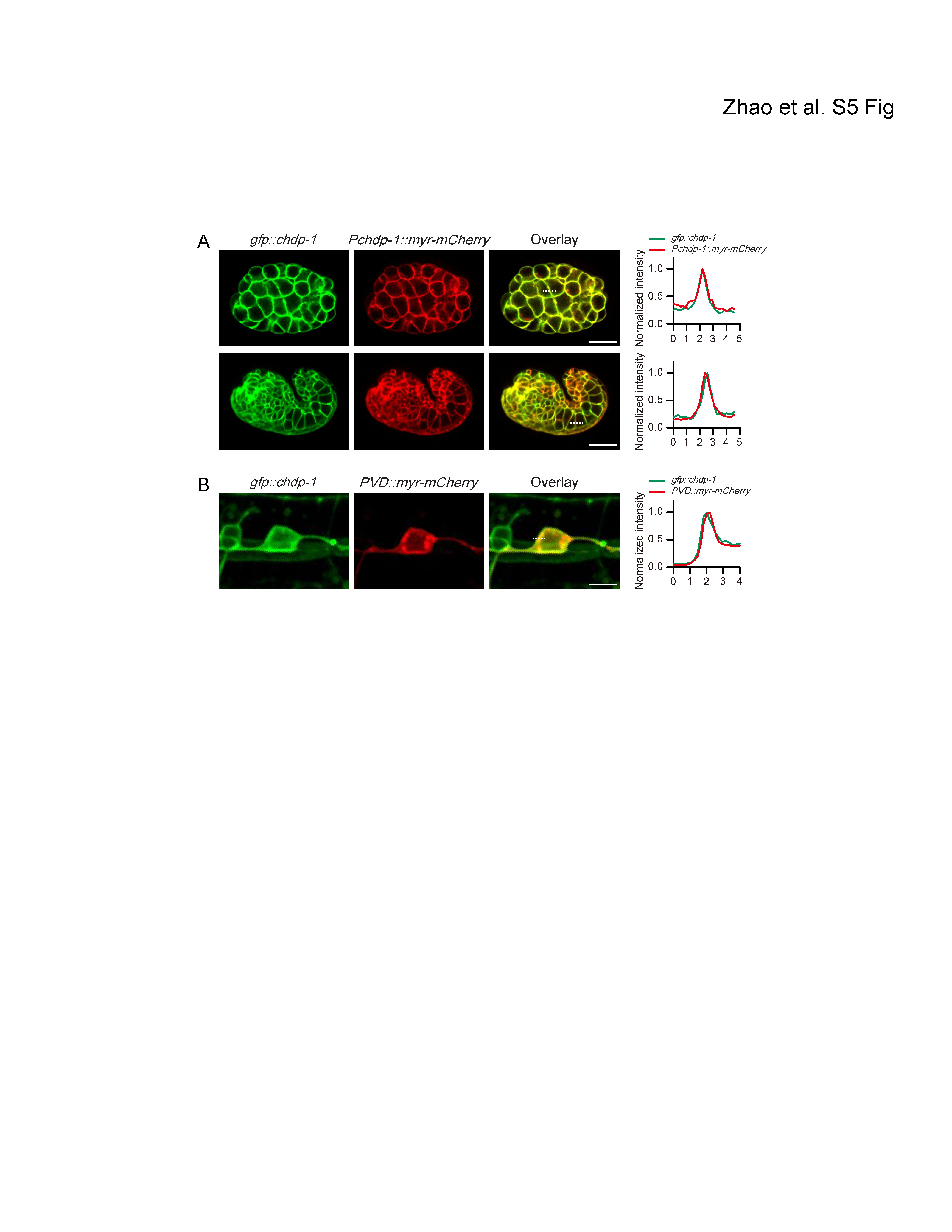

Supplement: S5 Fig — (A) Left: Confocal images showing the localization of endogenously expressed GFP::CHDP-1 (left), myr-mCherry (middle), and overlay (right) in the embryonic stages. Right: Normalized intensity of GFP::CHDP-1 and myr-mCherry around the cell membrane. Scale bar: 20 μm. (B) Left: Confocal images showing the localization of endogenously expressed GFP::CHDP-1 (left), myr-mCherry (middle), and overlay (right) in the PVD cell body. Right: Normalized intensity of GFP::CHDP-1 and myr-mCherry around the PVD cell body membrane. Scale bar: 5 μm. (TIFF) [file pgen.1010381.s005.tiff]

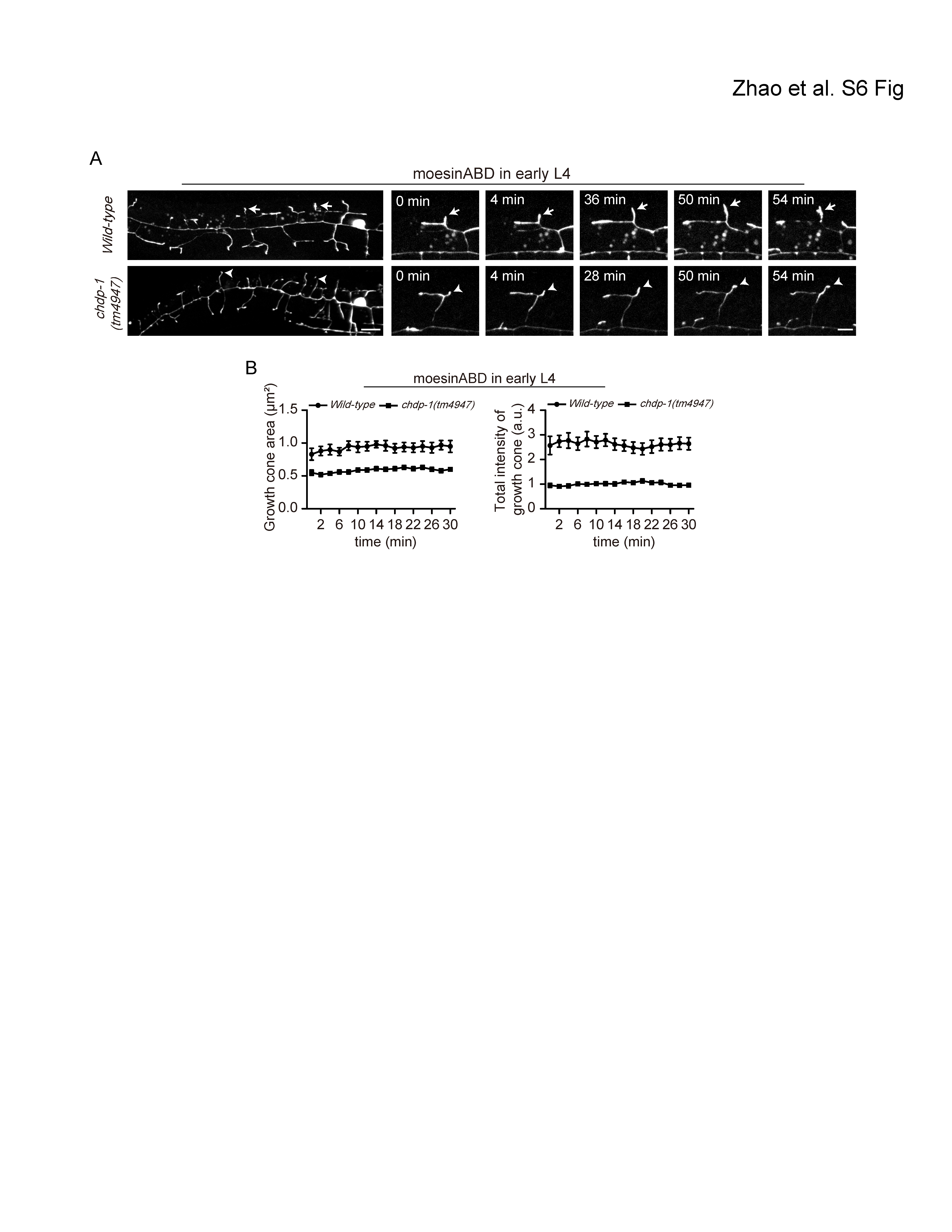

Supplement: S6 Fig — (A) Left: Confocal images of GFP::moesinABD in wild-type (upper), chdp-1(tm4947) mutant (lower) at the early L4 stage. Scale bar: 10 μm. Right: Confocal images from time-lapse movies of GFP::moesinABD in wild-type (upper) and chdp-1(tm4947) mutant (lower) during the early L4 stage. Arrows: growth cones in wild-type, arrowheads: growth cones in chdp-1(tm4947). Scale bar: 5 μm. (B) Quantification of the average growth cones area labeled by GFP::moesinABD, and the ratio of the intensity of the growth cones in the 4o branches to that of the primary dendrite in wild-type and chdp-1(tm4947) during the early L4 stage. n = 100 growth cones for each group. (TIFF) [file pgen.1010381.s006.tiff]

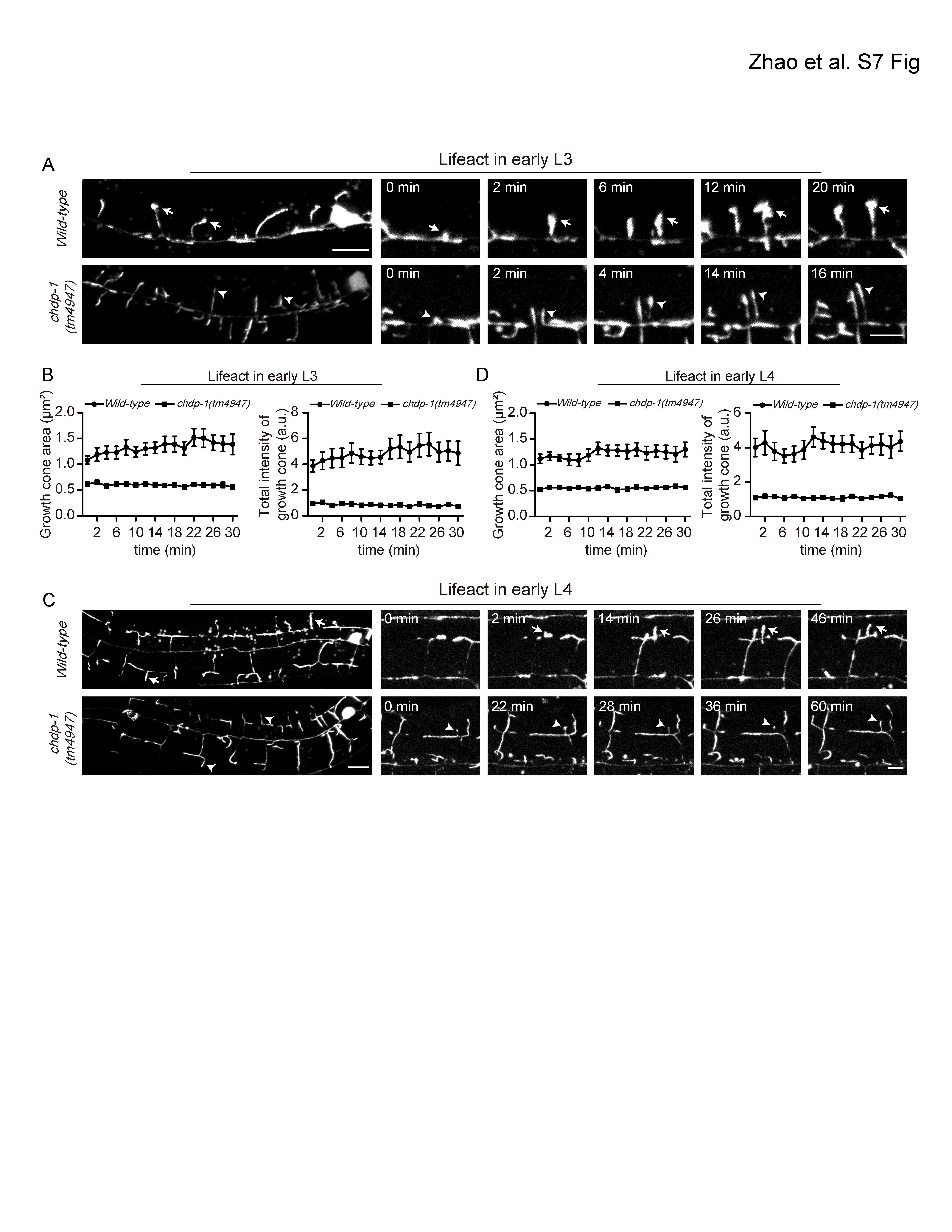

Supplement: S7 Fig — (A) Left: Confocal images of Lifeact::GFP in wild-type (upper) and chdp-1(tm4947) mutant (lower) at early L3 stage. Scale bar: 10 μm. Right: Confocal images from time-lapse movies showing actin assembly in wild-type (upper) and chdp-1(tm4947) mutant (lower) during the early L3 stage. Arrows: growth cones of 2o branches labeled by Lifeact::GFP in wild-type, arrowheads: growth cones in chdp-1(tm4947). Scale bar: 5 μm. (B) Quantification of the average growth cones area, and the ratio of the intensity in the growth cones of the 2o branches to that of the primary dendrite in wild-type and chdp-1(tm4947) during the early L3 stage. n = 100 growth cones for each group. (C) Left: Confocal images of Lifeact::GFP in wild-type (upper) and chdp-1(tm4947) mutant (lower) at early L4 stage. Scale bar: 10 μm. Right: Confocal images from time-lapse movies showing actin assembly in wild-type (upper) and chdp-1(tm4947) mutant (lower) during the early L4 stage. Arrows: growth cones of 4o branches labeled by Lifeact::GFP in wild-type, arrowheads: growth cones in chdp-1(tm4947). Scale bar: 5 μm. (D) Quantification of the average growth cones area, and the ratio of the intensity in the growth cones of the 4o branches to that of the primary dendrite in wild-type and chdp-1(tm4947) during the early L4 stage. n = 100 growth cones for each group. (TIFF) [file pgen.1010381.s007.tiff]

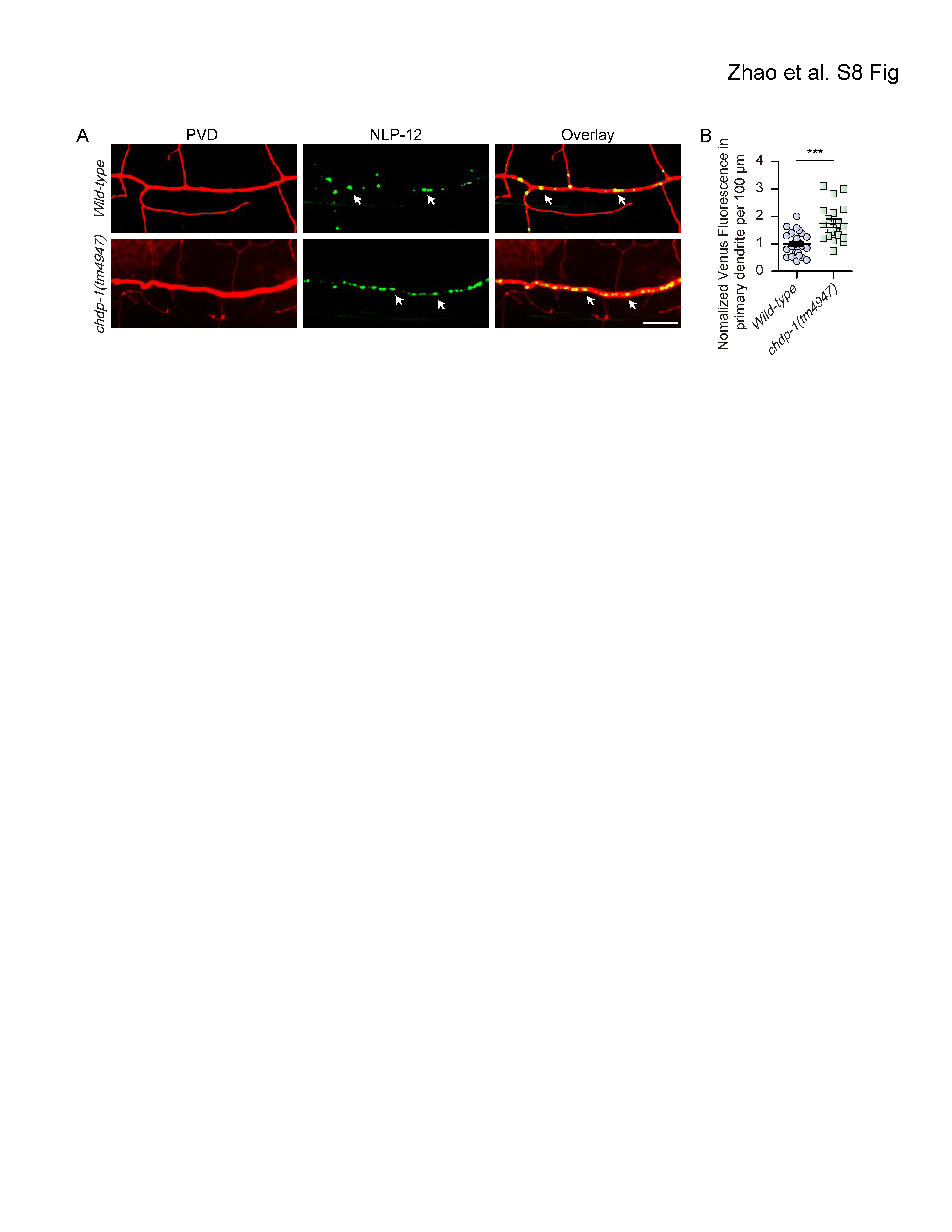

Supplement: S8 Fig — (A) Confocal images of PVD dendrites (left), NLP-12 (NLP-12::Venus) (middle), overlay (right) in wild-type (upper), chdp-1(tm4947) mutant (lower). Arrows: NLP-12::Venus-positive dense-core vesicles in primary dendrites. Scale bar: 10 μm. (B) Quantification of NLP-12::Venus fluorescence intensity in primary dendrites in a 100 μm area anterior to the PVD cell body (normalized to WT). Error bars: SEM. ***p < 0.001 by Student’s t test. n = 20–30 for each genotype. (TIFF) [file pgen.1010381.s008.tiff]

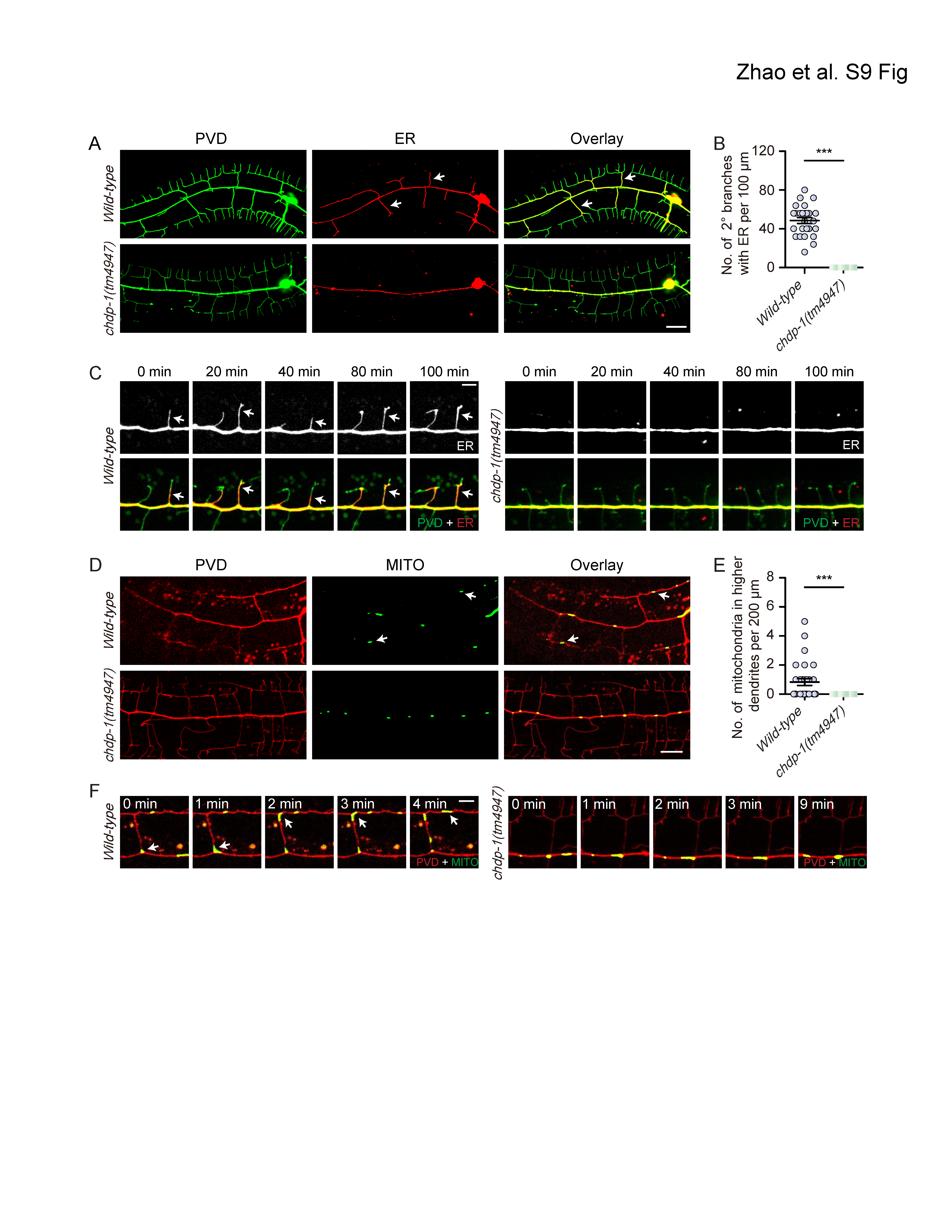

Supplement: S9 Fig — (A) Confocal images of PVD neuron (left), ER (labeled using a mCherry::SP12 reporter) (middle) and overlay (right) in wild-type (upper) and chdp-1(tm4947) mutant (lower). Arrows: ER in the high-ordered branches. Scale bar: 20 μm. (B) Quantification of the number of 2° branches with ER invasion in a 100 μm area anterior to the PVD cell body. Error bars: SEM. ***p < 0.001 by Student’s t-test. n = 20–30 for each genotype. (C) Confocal images from time-lapse movies showing transport of ER (upper) and overlay of ER (red) and PVD neuron (green) in wild-type (left) and chdp-1(tm4947) mutant (right) during early L3 stage. Arrows: ER in high-ordered branches. Scale bar: 5 μm. (D) Confocal images of PVD neuron (left), mitochondria (TOMM-20 1-54AA::GFP) (middle), overlay (right) in wild-type (upper) and chdp-1(tm4947) mutant (lower). Arrows: mitochondria in high-ordered branches. Scale bar: 20 μm. (E) Quantification of the number mitochondria in the high-ordered dendrites in a 200 μm area anterior to PVD cell body. Error bars: SEM. ***p < 0.001 by Student’s t test. n = 20–30 for each genotype. (F) Confocal images from time-lapse movies showing transport of mitochondria in PVD dendrites in wild-type (left) and chdp-1(tm4947) mutant (right) during L4 stage. Arrows: mitochondria in high-ordered branches. Scale bar: 5 μm. (TIFF) [file pgen.1010381.s009.tiff]

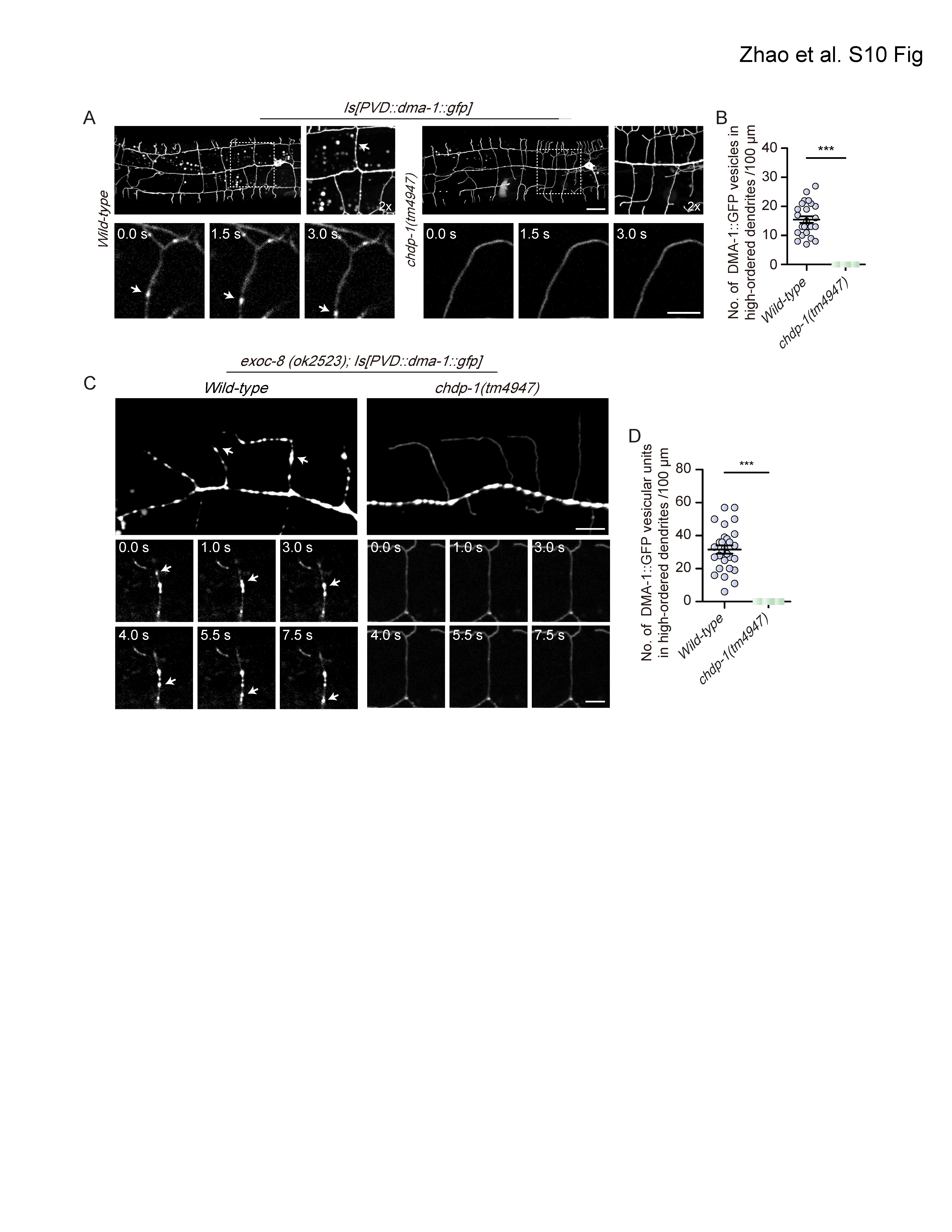

Supplement: S10 Fig — (A) Upper: Confocal images of DMA-1::GFP in wild-type (left) and chdp-1(tm4947) mutant (right). Scale bar: 20 μm. Lower: Confocal images from time-lapse movies showing transport of DMA-1::GFP vesicles in wild-type (left) and chdp-1(tm4947) mutant (right) at adult stages. Arrows: DMA-1::GFP vesicles moving in high-ordered branches. Scale bar: 5 μm. (B) Quantification of the number DMA-1::GFP vesicular units in the high-ordered dendrites in a 100 μm area anterior to the PVD cell body. Note that a DMA-1::GFP vesicular unit is defined as a punctum that is at least 2-fold brighter than the diffused signal along the dendrites. Error bars, SEM. ***p < 0.001 by Student’s t-test. n = 20–30 for each genotype. (C) Upper: Confocal images showing the distribution of DMA-1::GFP vesicular units in exoc-8 (ok2523) mutant background in wild-type (left) and chdp-1(tm4947) mutant (right). Scale bar: 20 μm. Lower: Confocal images from time-lapse movies showing transport of DMA-1::GFP vesicles in exoc-8 (ok2523) mutant background in wild-type (left) and chdp-1(tm4947) mutant (right) in adult. Arrows: DMA-1::GFP vesicles in the high-ordered branches. Note that loss of exoc-8 disrupts fusion between vesicles and the dendritic membranes, which make it easier to compare vesicular transport in wild-type and chdp-1 mutant animals. Scale bar: 5 μm. (D) Quantification of the number DMA-1::GFP vesicular units in the high-ordered dendrites in a 100 μm area anterior to the PVD cell body in exoc-8 (ok2523) background. Error bars, SEM. ***p < 0.001 by Student’s t-test. n = 20–30 for each genotype. (TIFF) [file pgen.1010381.s010.tiff]

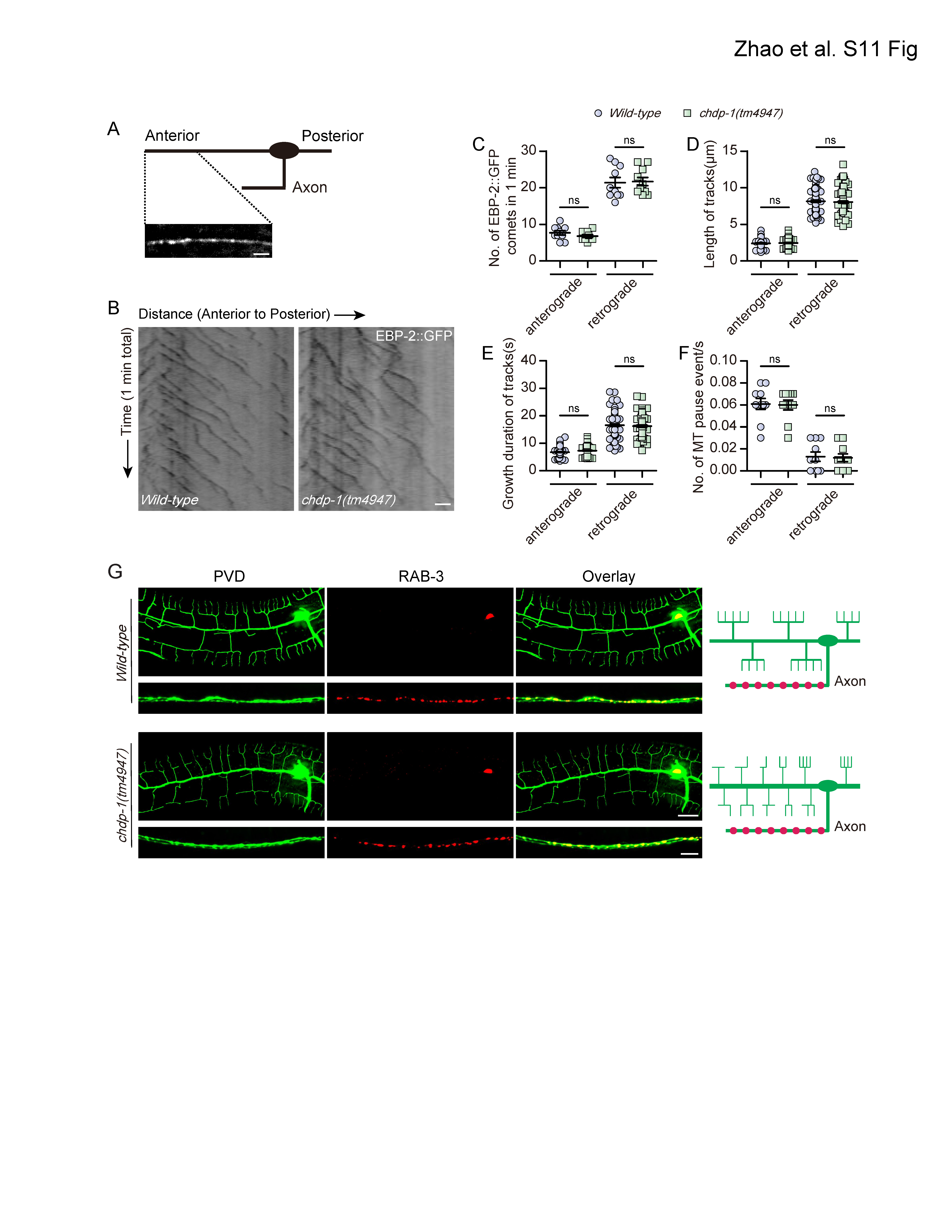

Supplement: S11 Fig — (A) A cartoon showing the area imaged to analyze the microtubule dynamics in the growth cone of the anterior primary dendrite. Scale bar: 2 μm. (B) Kymographs of EBP-2::GFP in a growth cone of the anterior primary dendrite in wild-type (left), chdp-1(tm4947) (right) during mid to late L2 stage. Scale bar: 2 μm. (C) Quantification of the number of EBP-2::GFP comets either moving away from the cell body (anterograde) or towards the cell body (retrograde) in wild-type and chdp-1(tm4947) mutant animals. Error bars, SEM. ns: non-significant by one-way ANOVA with the Tukey correction. n = 10 worms for each genotype. (D) Quantification of the length of tracks of the EBP-2 comets in wild-type and chdp-1(tm4947) mutant animals. Error bars, SEM. Ns: non-significant by one-way ANOVA with the Tukey correction. n = 10 worms for each genotype. (E) Quantification of the growth duration of tracks of the EBP-2 comets in wild-type and chdp-1(tm4947) mutant animals. Error bars, SEM. ns: non-significant by one-way ANOVA with the Tukey correction. n = 10 worms for each genotype. (F) Quantification of MT pause frequency in wild-type and chdp-1(tm4947) mutant animals. Error bars, SEM. ns: non-significant by one-way ANOVA with the Tukey correction. n = 10 worms for each genotype. (G) Left: Confocal images of PVD (top) dendrites and (down) axon (left), RAB-3 (middle), overlay (right) in wild-type (upper), chdp-1(tm4947) mutant (lower). Right: A cartoon showing the localization of RAB-3 in PVD dendrites and axon in wild-type and chdp-1(tm4947) mutants. Scale bar: 10 μm. (TIFF) [file pgen.1010381.s011.tiff]

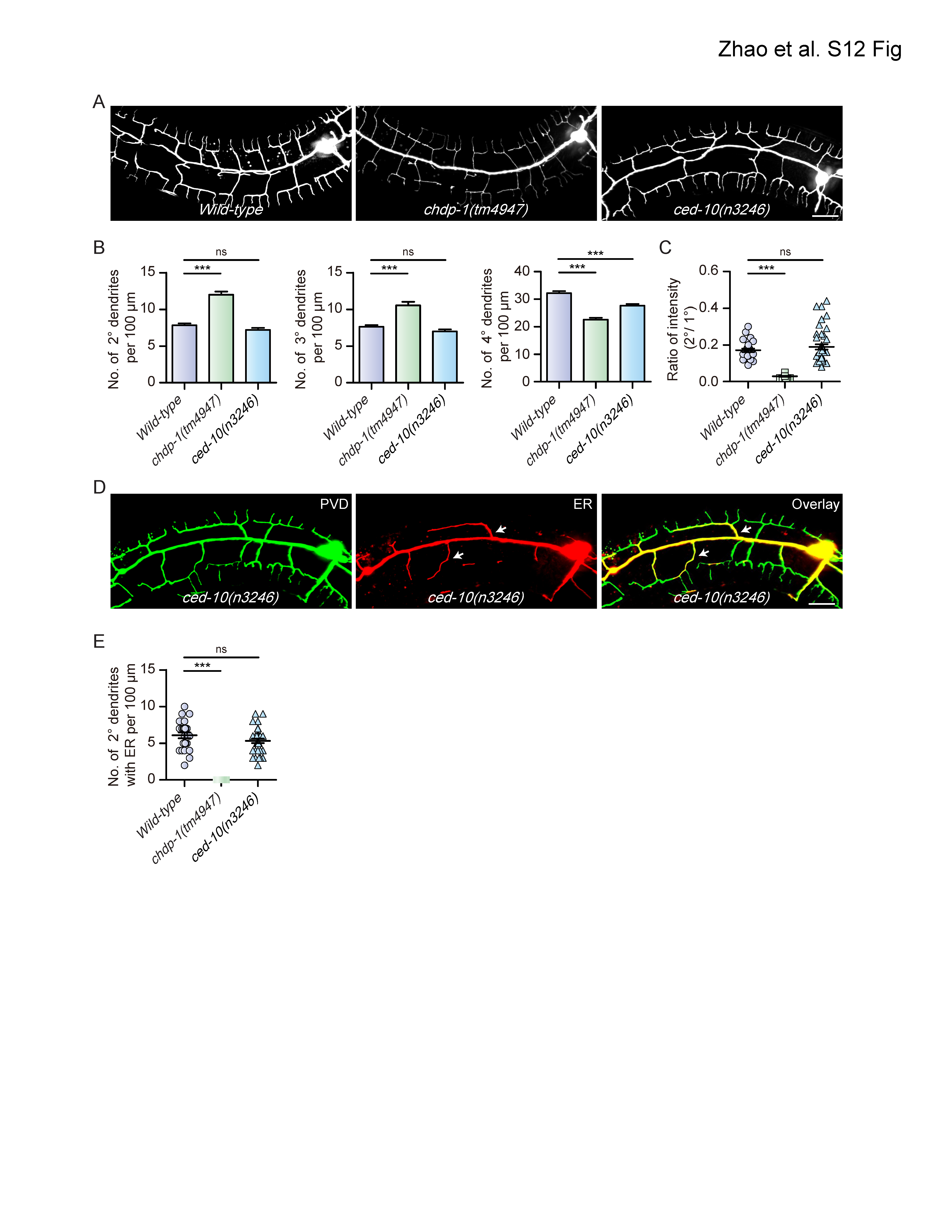

Supplement: S12 Fig — (A) Confocal images showing the PVD morphologies of wild-type, chdp-1(tm4947) and ced-10(n3246) mutant animals at 1DOA stage. Scale bar: 10 μm. (B-C) Quantification of (B) the number of 4o branches in a 100 μm area anterior to the PVD cell body, and (C) the ratio of the intensity in 2o branches to that of 1o dendrites for the genotypes indicated. Error bars, SEM. ***p < 0.001 by one-way ANOVA with the Tukey correction. ns: non-significant. n = 20–30 for each genotype. (D) Confocal images of PVD dendrites (left), ER (labeled using a mCherry::SP12 reporter) (middle), overlay (right) in ced-10(n3246) mutant. Arrows: ER in high-ordered branches. Scale bar: 20 μm. (E) Quantification of the number of 2° branches with ER invasion in wild-type, chdp-1(tm4947), ced-10(n3246) mutant in a 100 μm area anterior to the PVD cell body. Error bars, SEM. ***p < 0.001 by one-way ANOVA with the Tukey correction. ns: non-significant. n = 20–30 for each genotype. (TIFF) [file pgen.1010381.s012.tiff]

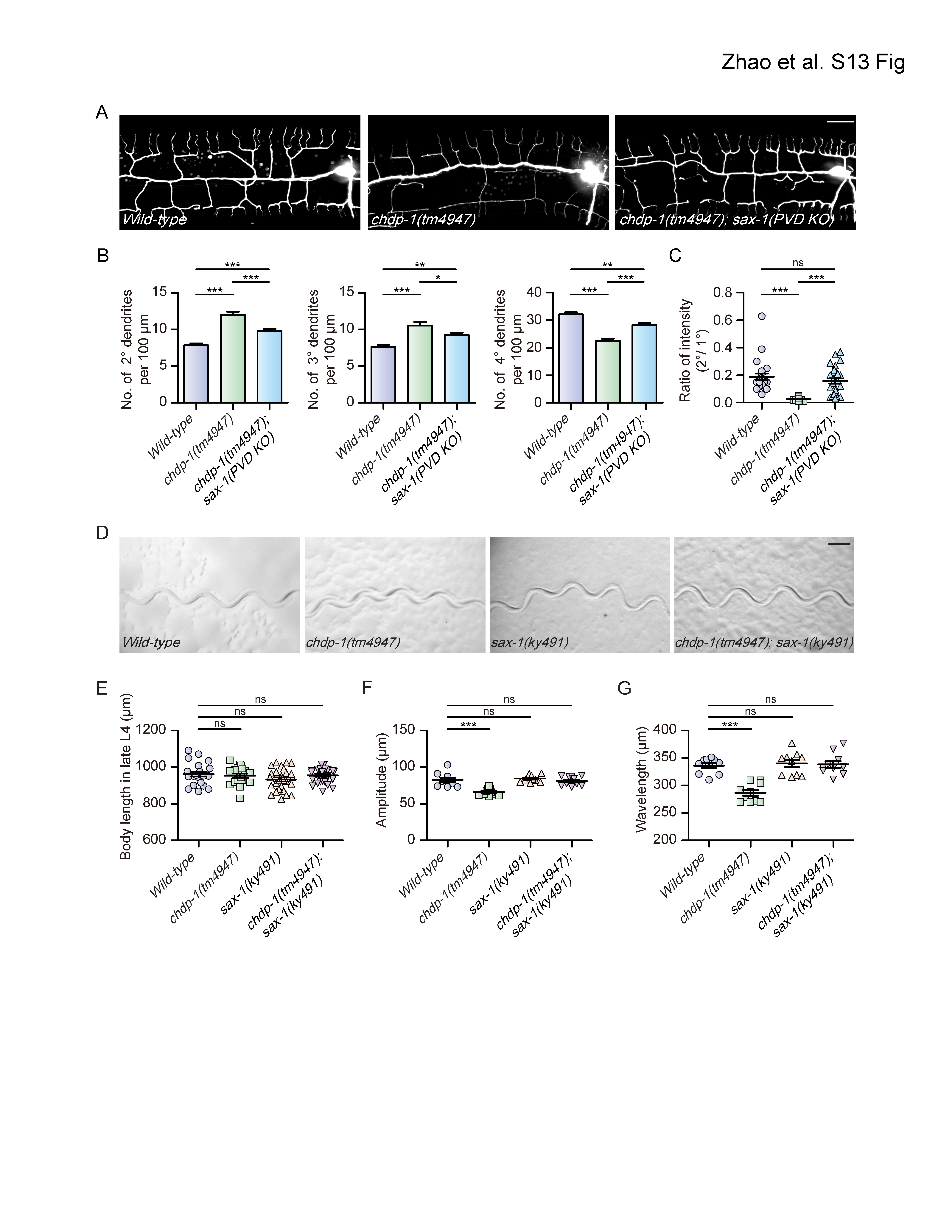

Supplement: S13 Fig — (A) Confocal images showing the PVD dendrite morphology of wild-type, chdp-1(tm4947) and chdp-1(tm4947); sax-1(PVD KO) mutant animals at the 1-day-old adult stage. Scale bar: 20 μm. (B-C) Quantification of (B) the number of 2o, 3o and 4o branches, and (C) the ratio of the intensity of the 2o branches to that of the primary dendrites in wild-type, chdp-1(tm4947) and chdp-1(tm4947); sax-1(PVD KO) in the 100 μm area anterior to PVD cell body. Error bars, SEM. *p < 0.05, **p < 0.01, ***p < 0.001 by one-way ANOVA with the Tukey correction. ns: non-significant. n = 20–40 for each genotype. (D) The representative moving tracks in wild-type, chdp-1(tm4947), sax-1(ky491) and chdp-1(tm4947); sax-1(ky491) mutants. Scale bar: 200 μm. (E-G) Quantification of the (E) body length, (F) amplitude and (G) wavelength of tracks in late L4 for the genotypes indicated. Error bars, SEM. ***p < 0.001 by one-way ANOVA with the Tukey correction. ns: non-significant. n = 10 worms for each genotype. (TIFF) [file pgen.1010381.s013.tiff]
