# Supplementary material for: The cell cortex-localized protein CHDP-1 is required for dendritic development and transport in C. elegans neurons
Source: PLoS Genet. 2022 Sep 20;18(9):e1010381. doi: 10.1371/journal.pgen.1010381 (PMC9524629; doi:10.1371/journal.pgen.1010381)
Supplement: S1 Table — (DOCX) [file pgen.1010381.s014.docx]

**S1 Table: Strains and plasmids used in this study**

| Identifiers | Description | Source | Additional information |
| --- | --- | --- | --- |
| TV15911 | *wyIs592[ser2prom3::myr-gfp+ Podr-1::rfp] III* | Kang Shen lab | To visualize PVD dendrite morphology |
| TV15921 | *wyIs594[ser2prom3::myr-gfp+ Podr-1::rfp] V* | Kang Shen lab | To visualize PVD dendrite morphology |
| GSW5063 | *chdp-1(zac135) I; wyIs592* | This study | To visualize PVD dendrite morphology in *chdp-1(zac135)* |
| GSW5126 | *chdp-1(tm4947) I; wyIs592* | This study | To visualize PVD dendrite morphology in *chdp-1(tm4947)* |
| GSW6011 | *chdp-1(zac278[gfp::chdp-1]) I* | This study | To visualize the endogenous expression of CHDP-1 |
| TV15919 | *wyIs587[ser2prom3::myr-gfp+ Podr-1::rfp] II* | Kang Shen lab | To visualize PVD dendrite morphology |
| GSW6491 | *chdp-1(zac278[gfp::chdp-1]) I; wyIs587* | This study | To detect whether *gfp* knock-in affects the function of CHDP-1 |
| GSW5605 | *chdp-1(tm4947);*  *wyIs592;*  *zacEx301[Pchdp-1::chdp-1]* | This study | A positive control for the tissue-specific rescue experiments |
| GSW5172 | *chdp-1(tm4947);*  *wyIs592;*  *zacEx29[ser2prom3::chdp-1]* | This study | To determine whether CHDP-1 acts in the PVD neurons |
| GSW5609 | *chdp-1(tm4947);*  *wyIs592;*  *zacEx305[Pdpy-7::chdp-1]* | This study | To determine whether CHDP-1 acts in the epidermis |
| GSW5810 | *chdp-1(tm4947);*  *wyIs592; zacEx381[Phlh-1::chdp-1]* | This study | To determine whether CHDP-1 acts in the body wall muscle cells |
| GSW6921 | *chdp-1(tm4947); wyIs592; zacTi22[Phsp-16.48::chdp-1::sl2::bfp]* | This study | To determine when CHDP-1 acts to regulate dendrite development |
| GSW5544 | *chdp-1(tm4947); wyIs592; zacEx261[ser2prom3::chdp-1 ΔP1]* | This study | To explore whether CHDP-1 lacking the P1 domain can function normally |
| GSW5547 | *chdp-1(tm4947); wyIs592; zacEx264[ser2prom3::chdp-1 ΔP2]* | This study | To explore whether CHDP-1 lacking the P2 domain can function normally |
| GSW5551 | *chdp-1(tm4947); wyIs592; zacEx268[ser2prom3::chdp-1 ΔCH]* | This study | To explore whether CHDP-1 lacking the CH domain can function normally |
| GSW5555 | *chdp-1(tm4947); wyIs592; zacEx272[ser2prom3::chdp-1 ΔHelix]* | This study | To explore whether CHDP-1 lacking the helix domain can function normally |
| GSW5557 | *chdp-1(tm4947); wyIs592; zacEx274[ser2prom3::chdp-1 ΔC]* | This study | To explore whether CHDP-1 lacking the C terminal part can function normally |
| GSW5405 | *zacEx179[ser2prom3::gfp::chdp-1]* | This study | To determine CHDP-1 subcellular localization in the PVD neurons |
| GSW5792 | *zacEx365[ser2prom3::gfp::chdp-1 ΔP1]* | This study | To explore whether CHDP-1 lacking the P1 domain can be expressed and localized normally |
| GSW5797 | *zacEx370[ser2prom3::gfp::chdp-1 ΔP2]* | This study | To explore whether CHDP-1 lacking the P2 domain can be expressed and localized normally |
| GSW5800 | *zacEx373[ser2prom3::gfp::chdp-1 ΔCH]* | This study | To explore whether CHDP-1 lacking the CH domain can be expressed and localized normally |
| GSW5793 | *zacEx366[ser2prom3::gfp::chdp-1 ΔHelix]* | This study | To explore whether CHDP-1 lacking the Helix domain can be expressed and localized normally |
| GSW5803 | *zacEx376[ser2prom3::gfp::chdp-1 ΔC]* | This study | To explore whether CHDP-1 lacking the C terminal can be expressed and localized normally |
| GSW5834 | *zacEx398[ser2prom3::gfp]* | This study | To label the PVD dendrites with a cytosolic GFP reporter |
| GSW6572 | *chdp-1(zac283[gfp11(7x)::chdp-1]) I.* | This study | To visualize the endogenous expression of CHDP-1 specifically in the PVD neuron neurons |
| GSW6715 | *chdp-1(zac283[gfp11(7x)::chdp-1]) I; zacEx1130[ser2prom3::gfp(1-10)]* | This study | To visualize the endogenous expression of CHDP-1 in the PVD neurons |
| GSW6718 | *chdp-1(zac283[gfp11(7x)::chdp-1]) I; zacEx1133[ser2prom3::gfp(1-10)+ ser2p3:: mcherry::moesinABD]* | This study | Co-labeling of the endogenous CHDP-1 and F-actin in the dendritic growth cones |
| GSW5527 | *zacEx250[ser2prom3::gfp ::moesinABD]* | This study | To visualize the dynamic of F-actin during PVD dendrite growth in wild-type |
| GSW5577 | *chdp-1(tm4947); zacEx250[ser2prom3::gfp ::moesinABD]* | This study | To visualize the dynamic of F-actin during PVD dendrite growth in *chdp-1(tm4947)* |
| TV17245 | *dma-1(wy686) I ;*  *wyIs592* | Kang Shen lab | To visualize PVD dendrite morphology in *dma-1(wy686)* |
| GSW7477 | *zacEx1639[ser2prom3::nlp-12::Venus::SL2::mcherry]* | This study | To visualize the NLP-12-positive dense-core vesicles in PVD in wild-type |
| GSW7488 | *chdp-1(tm4947); zacEx1642[ser2prom3::nlp-12::Venus::SL2::mcherry]* | This study | To visualize the NLP-12-positive dense-core vesicles in PVD in *chdp-1(tm4947)* |
| GSW7491 | *sax-7(nj48);*  *zacEx1645[ser2prom3::nlp-12::Venus::SL2::mcherry+Pdpy-7::vhhGFP4::sax-7]* | This study | To visualize NLP-12::Venus secretion (captured by the epidermis-localized GBP::SAX-7) |
| GSW7497 | *chdp-1(tm4947);*  *zacEx1651[ser2prom3::nlp-12::Venus::SL2::mcherry+ Pdpy-7:: vhhGFP4::sax-7]* | This study | To visualize NLP-12::Venus secretion (captured by the epidermis-localized GBP::SAX-7) |
| GSW6909 | *zacEx1279[ser2prom3::gfp::tba-1]* | This study | To visualize TBA-1 in PVD in wild-type |
| GSW6973 | *chdp-1(tm4947); zacEx1279[ser2prom3::gfp::tba-1]* | This study | To visualize TBA-1 in PVD in *chdp-1(tm4947)* |
| GSW6915 | *zacEx1285[ser2prom3::ebp-2::gfp]* | This study | To visualize the dynamic of EBP-2 in PVD dendrites in wild-type animals |
| GSW6974 | *chdp-1(tm4947); zacEx1285[ser2prom3::ebp-2::gfp]* | This study | To visualize the dynamic of EBP-2 in PVD dendrites of *chdp-1(tm4947)* animals |
| GSW7522 | *sax-1(ky491) X;*  *wyIs592* | This study | To visualize PVD dendrite morphology in *sax-1(ky491)* |
| GSW7515 | *chdp-1(tm4947);*  *sax-1(ky491);*  *wyIs592* | This study | To visualize PVD dendrite morphology in *chdp-1(tm4947); sax-1(ky491)* |
| GSW7588 | *sax-2(ky216);*  *wyIs594* | This study | To visualize PVD dendrite morphology in *sax-2(ky216)* |
| GSW7607 | *chdp-1(tm4947);*  *sax-2(ky216);*  *wyIs594* | This study | To visualize PVD dendrite morphology in *chdp-1(tm4947); sax-2(ky216)* |
| GSW5567 | *zacEx280[ser2prom3::lifeact::gfp]* | This study | To visualize the dynamic of F-actin during PVD dendrite growth in wild-type |
| GSW5666 | *chdp-1(tm4947); zacEx280[ser2prom3::lifeact::gfp]* | This study | To visualize the dynamic of F-actin during PVD dendrite growth in *chdp-1(tm4947)* |
| GSW7604 | *sax-1(ky491); zacEx280[ser2prom3::lifeact::gfp]* | This study | To visualize the dynamic of F-actin during PVD dendrite growth in *sax-1(ky491)* |
| GSW7605 | *chdp-1(tm4947);*  *sax-1(ky491); zacEx280[ser2prom3::lifeact::gfp]* | This study | To visualize the dynamic of F-actin during PVD dendrite growth in *chdp-1(tm4947)*  *; sax-1(ky491)* |
| GSW7545 | *sax-1(ky491); zacEx1279[ser2prom3::gfp::tba-1]* | This study | To visualize TBA-1 in PVD dendrites in *sax-1(ky491)* |
| GSW7587 | *chdp-1(tm4947);*  *sax-1(ky491); zacEx1279[ser2prom3::gfp::tba-1]* | This study | To visualize TBA-1 in PVD dendrites in *chdp-1(tm4947); sax-1(ky491)* |
| GSW7543 | *sax-1(ky491); zacEx1639[ser2prom3::nlp-12::Venus::SL2::mcherry]* | This study | To visualize the NLP-12-positive dense-core vesicles in PVD dendrites in *sax-1(ky491)* |
| GSW7582 | *chdp-1(tm4947);*  *sax-1(ky491); zacEx1639[ser2prom3::nlp-12::Venus::SL2::mcherry]* | This study | To visualize the NLP-12-positive dense-core vesicles in PVD dendrites in *chdp-1(tm4947); sax-1(ky491)* |
| NC1686 | *wdIs51[F49H12.4::gfp, unc-119(+)] X* | CGC/ David Miller lab | To visualize PVD dendrite morphology |
| GSW5084 | *chdp-1(zac135);*  *wdIs51* | This study | To visualize PVD dendrite morphology in *chdp-1(zac135)* |
| GSW5260 | *chdp-1(tm4947);*  *wdIs51* | This study | To visualize PVD dendrite morphology in *chdp-1(tm4947)* |
| OH1422 | *otIs138[ser2prom3::gfp, rol-6(d)] X* | CGC/Oliver Hobert lab | To visualize PVD dendrite morphology |
| GSW5082 | *chdp-1(zac135);*  *otIs138* | This study | To visualize PVD dendrite morphology in *chdp-1(zac135)* |
| GSW5196 | *chdp-1(tm4947);*  *otIs138* | This study | To visualize PVD dendrite morphology in *chdp-1(tm4947)* |
| GSW7979 | *chdp-1(zac283[gfp11(7x)::chdp-1]);zacEx1907[ser2prom3::gfp(1-10)+ser2prom3::myr-mcherry]* | This study | To detect whether *gfp(7x)* knock-in affects the function of CHDP-1 |
| GSW7976 | *chdp-1(zac278[gfp::chdp-1]);*  *zacEx1904[Pchdp-1::myr-mcherry]* | This study | Co-labeling of endogenous CHDP-1 and a cell membrane marker |
| GSW7982 | *chdp-1(zac278[gfp::chdp-1]);*  *zacEx1910[ser2prom3::myr-mcherry]* | This study | Co-labeling of endogenous CHDP-1 and a PVD plasma membrane marker |
| GSW5407 | *wyIs592; zacEx181[ser2prom3::mcherry::sp12]* | This study | To visualize the ER in PVD dendrites in wild-type |
| GSW5431 | *chdp-1(tm4947); wyIs592; zacEx181[ser2prom3::mcherry::sp12]* | This study | To visualize the ER in PVD dendrites in *chdp-1(tm4947)* |
| GSW5041 | *wyIs50063[ser2prom3::mito::gfp::ser2prom3::myr-mcherry]* | This study | To visualize PVD dendrite morphology and mitochondria in PVD dendrites in wild-type |
| GSW5214 | *chdp-1(tm4947); wyIs50063[ser2prom3::mito::gfp::ser2prom3::myr-mcherry]* | This study | To visualize PVD dendrite morphology and mitochondria in PVD dendrites in *chdp-1(tm4947)* |
| TV20130 | *wyIs740[ser2prom3::dma-1::gfp+ Podr-1::gfp] V* | Kang Shen lab | To visualize the subcellular localization and dynamic transport of DMA-1 in PVD dendrites in wild-type |
| GSW5208 | *chdp-1(tm4947);*  *wyIs740[ser2prom3::dma-1::gfp+ Podr-1::gfp]* | This study | To visualize the subcellular localization and dynamic transport of DMA-1 in PVD dendrites in *chdp-1(tm4947)* |
| GSW6101 | *exoc-8(ok2523);*  *wyIs740[ser2prom3::dma-1::gfp+ Podr-1::gfp]* | This study | To visualize the subcellular localization and dynamic transport of DMA-1 in PVD dendrites in *exoc-8(ok2523)* |
| GSW6121 | *exoc-8(ok2523);*  *chdp-1(tm4947);*  *wyIs740[ser2prom3::dma-1::gfp+ Podr-1::gfp]* | This study | To visualize the subcellular localization and dynamic transport of DMA-1 in PVD dendrites in *exoc-8(ok2523); chdp-1(tm4947)* |
| GSW8046 | *wyIs592; zacEx1969[Pdes-2::mcherry::rab-3]* | This study | To visualize the distribution of RAB-3 in PVD neurons in *wild-type* |
| GSW8086 | *chdp-1(tm4947); wyIs592; zacEx1969[Pdes-2::mcherry::rab-3]* | This study | To visualize the distribution of RAB-3 in PVD neurons in *chdp-1(tm4947)* |
| GSW5393 | *ced-10(n3246) IV;*  *wyIs592* | This study | To visualize PVD dendrite morphology in *ced-10(n3246)* |
| GSW6415 | *ced-10(n3246); wyIs592;*  *zacEx181[ser2prom3::mcherry::sp12]* | This study | To visualize the ER in PVD dendrites in *ced-10(n3246)* |
| GSW7520 | *chdp-1(tm4947);*  *zacEx1667[Pnhr-81::cas9+ sax-1 sgRNA 1,2,3]* | This study | To knockout *sax-1* in PVD in *chdp-1(tm4947)* mutants |
| pZT74 | *chdp-1 left HA::gfp::* *chdp-1 right HA* | This study | Template to make gfp knock-in into the endogenous *chdp-1* locus |
| pZT72 | *pU6::chdp-1 sgRNA 1* | This study | SgRNA for *gfp::chdp-1* knock-in |
| pZT73 | *pU6::chdp-1 sgRNA 2* | This study | SgRNA for *gfp::chdp-1* knock-in |
| pZT71 | *pU6::chdp-1 sgRNA 3* | This study | SgRNA for *gfp::chdp-1* knock-in |
| pSX524 | *Peft-3::cas9 + PU6::dpy-10 sgRNA* | Suhong Xu lab | To express Cas9 in germ cells for CRISPR knock-in |
| pZT6 | *Pchdp-1::chdp-1* | This study | To express CHDP-1 under its own promoter |
| pZT3 | *ser2prom3::chdp-1* | This study | To express CHDP-1 in the PVD neurons |
| pZT4 | *Pdpy-7::chdp-1* | This study | To express CHDP-1 in the epidermis |
| pZT5 | *Phlh-1::chdp-1* | This study | To express CHDP-1 in the body wall muscles |
| pZT116 | *miniMos Phsp-16.48::chdp-1::sl2::bfp* | This study | To make a single copy transgene to express CHDP-1 driven by a heat-shock promoter |
| pCFJ601 | P*eft-3:mos1 transposase:tbb-2* UTR | Erik Jorgensen lab | To express the mos1 transposase in germ cells for generating single copy transgenes |
| pZT52 | *ser2prom3::chdp-1 ∆ P1* ( *∆*18-38 amino acids) | This study | For structure-function analysis |
| pZT53 | *ser2prom3::chdp-1 ∆ P2* ( *∆*95-104 amino acids) | This study | For structure-function analysis |
| pZT54 | *ser2prom3::chdp-1 ∆ CH* ( *∆*121-247 amino acids) | This study | For structure-function analysis |
| pZT55 | *ser2prom3::chdp-1 ∆ Helix* ( *∆*253-282 amino acids) | This study | For structure-function analysis |
| pZT56 | *ser2prom3::chdp-1 ∆ C* ( *∆*283-338 amino acids) | This study | For structure-function analysis |
| pZT58 | *ser2prom3::gfp::chdp-1* | This study | To examine the subcellular localization of the full length CHDP-1 |
| pZT64 | *ser2prom3::gfp::chdp-1 ∆ P1* ( *∆*18-38 amino acids) | This study | To examine the subcellular localization of the truncated CHDP-1 |
| pZT65 | *ser2prom3::gfp::chdp-1 ∆ P2* ( *∆*95-104 amino acids) | This study | To examine the subcellular localization of the truncated CHDP-1 |
| pZT66 | *ser2prom3::gfp::chdp-1 ∆ CH* ( *∆*121-247 amino acids) | This study | To examine the subcellular localization of the truncated CHDP-1 |
| pZT67 | *ser2prom3::gfp::chdp-1 ∆ Helix* ( *∆*253-282 amino acids) | This study | To examine the subcellular localization of the truncated CHDP-1 |
| pZT68 | *ser2prom3::gfp::chdp-1 ∆ C* ( *∆*283-338 amino acids) | This study | To examine the subcellular localization of the truncated CHDP-1 |
| pZT105 | *chdp-1 left HA::gfp11(7x)::chdp-1 right HA* | This study | Template for knock-in *gfp11(7x)*::*chdp-1* |
| pZT106 | *ser2prom3::spGFP(1-10)* | This study | To express spGFP(1-10) in PVD neurons |
| pWZ11 | *ser2prom3::gfp* | This study | To label the PVD neurons |
| pZT77 | *ser2prom3::mcherry::moesinABD* | This study | To label F-actin in the PVD neurons |
| pXD200 | *ser2prom3::gfp::moesinABD* | Kang Shen lab | To label F-actin in the PVD neurons |
| pZT143 | *ser2prom3::nlp-12::Venus::SL2::mcherry* | This study | To label NLP-12 with Venus in the PVD neurons |
| pTL110 | *Pdpy-7::vhhGFP4::sax-7* | Kang Shen lab | To capture NL-12::Venus secreted from the PVD dendrites |
| pZT117 | *ser2prom3::gfp::tba-1* | This study | To label the microtubule cytoskeleton in the PVD neurons |
| pZT119 | *ser2prom3::ebp-2::gfp* | This study | To label the plus-end of the microtubules in the PVD neurons |
| pZT59 | *ser2prom3::lifeact::gfp* | This study | To label F-actin in the PVD neurons |
| pZT177 | *ser2prom3::myr-mcherry* | This study | To label the PVD cell membranes |
| pZT190 | *Pchdp-1::myr-mcherry* | This study | To label the cell membranes |
| pZT37 | *ser2prom3::mcherry::sp12* | This study | To label ER in the PVD neurons |
| pZT193 | *Pdes-2::mcherry::rab-3* | This study | To label RAB-3 in the PVD neurons |
| pWZ243 | *Pnhr-81::cas9* | Kang Shen lab | To express Cas9 in the seam cells to knock-out genes in PVD and other cells derived from the seam cell lineage |
| pZT146 | *pU6:: sax-1 sgRNA 1* | This study | To express sgRNA for knock-out *sax-1* in PVD |
| pZT147 | *pU6:: sax-1 sgRNA 2* | This study | To express sgRNA for knock-out *sax-1* in PVD |
| pZT148 | *pU6:: sax-1 sgRNA 3* | This study | To express sgRNA for knock-out *sax-1* in PVD |
| pCFJ90 | *Pmyo-2::mcherry* | Erik Jorgensen lab | Co-injection marker |
| pCFJ104 | *Pmyo-3::mcherry* | Erik Jorgensen lab | Co-injection marker |
| N/A | *Podr-1::rfp* | Kang Shen lab | Co-injection marker |
| N/A | *Punc-122::rfp* | Kang Shen lab | Co-injection marker |
