# Supplementary material for: The cell cortex-localized protein CHDP-1 is required for dendritic development and transport in C. elegans neurons
Source: PLoS Genet. 2022 Sep 20;18(9):e1010381. doi: 10.1371/journal.pgen.1010381 (PMC9524629; doi:10.1371/journal.pgen.1010381)
Supplement: Attachment — Submitted filename: Response to the review comments.pdf [file pgen.1010381.s016.pdf]

Dear Dr. Chisholm,

Thank you for handling our manuscript PGENETICS-D-22-00369, “CHDP-1-dependent cortical actin assembly is required for dendritic development and transport in *C. elegans* neurons”. We also thank all the reviewers for their insightful comments and constructive suggestions. Responding to the reviewers’ comments, we have performed the following additional experiments/ quantifications.

(1) We performed confocal imaging analysis and found that the endogenous CHDP-1 colocalizes with the plasma membrane marker myr-mCherry both in cells of the embryos and the PVD cell bodies. However, due to the limited resolution of the confocal imaging system and the small diameter of PVD dendrites, we were not able to tell whether CHDP-1 is localized on the cell cortex of the PVD dendrites.

(2) We quantified the number of 2° and 4° branches in both WT and *chdp-1* mutants. We found that in addition to the degeneration of the primary dendrites, the number of 2° branches is also decreased in adult *chdp-1* mutant animals at day 7 and day 9.

(3) We measured the intensity of GFP(7x)::CHDP-1 in the growth cones of both the primary dendrites and 2° branches. CHDP-1 is indeed enriched in the growth cones as indicated by the quantifications.

(4) We quantified the number of 2°, 3° and 4° branches in *gfp(7x)::chdp-1* knock-in animals and found that there was no defect in dendrite morphogenesis. These data suggest that attaching the seven consecutive GFP11 modules to the endogenous CHDP-1 does not affect its function in dendrite development.

(5) We quantified the percentage of palm-shaped and finger-shaped growth cones in both WT and *chdp-1* mutant animals. We found that most of the 2° growth cones are palm-shaped in WT animals, while most of them are finger-shaped in *chdp-1* mutant animals.

(6) We quantified the number of NLP-12::Venus puncta in the primary PVD dendrites and found that indeed there are about 2-fold more puncta in the *chdp-1* mutant animals.

(7) We generated a new strain to co-label both CHDP-1 and mCherry::TBA-1 and found that these two proteins show co-localization around the cell cortex, indicating that CHDP-1 might be associated with the microtubule cytoskeleton.

(8) To explore whether CHDP-1 genetically interacts with players in actin disassembly, we over-expressed UNC-60 in both WT and *chdp-1* mutant genetic background. Interestingly, over-expression of UNC-60 partially restored the intensity of 2º and 3º branches.

(9) We also knock-down *nmy-1* and *nmy-2* by RNAi and somatic CRISPR. We did not find any similar defect as that of the *chdp-1* mutant animals.

(10) We treated WT and *chdp-1* mutant animals with colchicine or taxol. We did not see any enhanced or suppressed phenotypes for dendrite development in *chdp-1* mutant background.

(11) We quantified the growth duration and length of the EBP-2 tracks in the primary dendrite growth cones, and checked the pause frequency. We did not see any differences between the WT and the *chdp-1* mutant animals. We also made a mCherrry::RAB-3 reporter to examine whether there is any mis-localization of RAB-3 in dendrites of *chdp-1* mutants. We did not see any mis-localization, suggesting that the microtubule polarity of the primary dendrites in the *chdp-1* mutant animals is normal.

(12) We generated a SAX-1::GFP knock-in strain by CRISPR and compared the expression levels in WT and *chdp-1* knock-out animals. We did not see any difference, suggesting that the amount of SAX-1 is not upregulated in *chdp-1* mutants.

We have also changed wordings for some of our interpretations as suggested by the reviewers, and added more information in the materials and methods part. We hope that our revised manuscript will now be suitable for publication in Plos Genetics. Please let me know if there are additional questions or concerns regarding our submission.

Reviewer #1: Review PGENETICS-D-22-00369 (Zhao…. Wei Zou)

Zhao et al. described the role of the cell cortex protein CHDP-1 in shaping the dendritic architecture of the C. elegans multidendritic neuron PVD. Through extensive genetic and imaging investigations, they showed that CHDP-1 controls the morphology of higher order PVD dendrites,

promotes actin and microtubule assembly and facilitates the transport of the NLP-12 neuropeptide as well as organelles such as mitochondria and ER. All these likely translate into maintaining an intact PVD function for proprioception, as the chdp-1 mutant displays locomotion features of defective proprioception, including shallower and shorter waveforms in its sinusoidal movement. Interestingly, mutations in the sax-1 kinase gene suppressed the defects in PVD dendrite morphology, actin assembly and proprioceptive functions of the chdp-1 mutant, suggesting that SAX-1 antagonizes CHDP-1 functions. In general, the experiments were well planned and rigorously executed, and most of the results were convincing. Below I list a few concerns that the authors should address before the paper can be considered for publication in PLoS Genetics.

We thank this review for the positive comments on our manuscript.

Major comments:

1. A key point in the paper is the localization of CHDP-1 to the cell cortex. However, the regular confocal microscopy used in Figure 4 is unlikely to provide the spatial resolution required to document CDHP-1 localization to the cell cortex. The authors should attempt STED super-resolution microscopy of GFP::CHDP-1 with an appropriate membrane marker such as myristoylated mCherry.

We thank the reviewer for this suggestion. In a previous study, we reported that GFP::CHDP-1 expressed from a transgene co-localizes with the plasma membrane marker myr-mCherry in the cell bodies of PLM neurons (Guan et al., 2016, Plos Genet). To determine whether the endogenous CHDP-1 also localizes to the cell cortex, we co-expressed myr-mCherry in this strain. As the results showed in S5 Fig, these two proteins display co-localization. These data suggest that CHDP-1 is a cell cortex-localized protein. We do acknowledge that we have no evidence to show that CHDP-1 localizes to the cell cortex of the PVD dendrites. However, previously we performed STED super-resolution microscopy of myr-GFP and we did not see good cell membrane localization, probably due to the small diameter of the PVD dendrites. So, the STED microscopy might not be good enough to examine whether CHDP-1 localizes to the cell cortex of PVD dendrites.

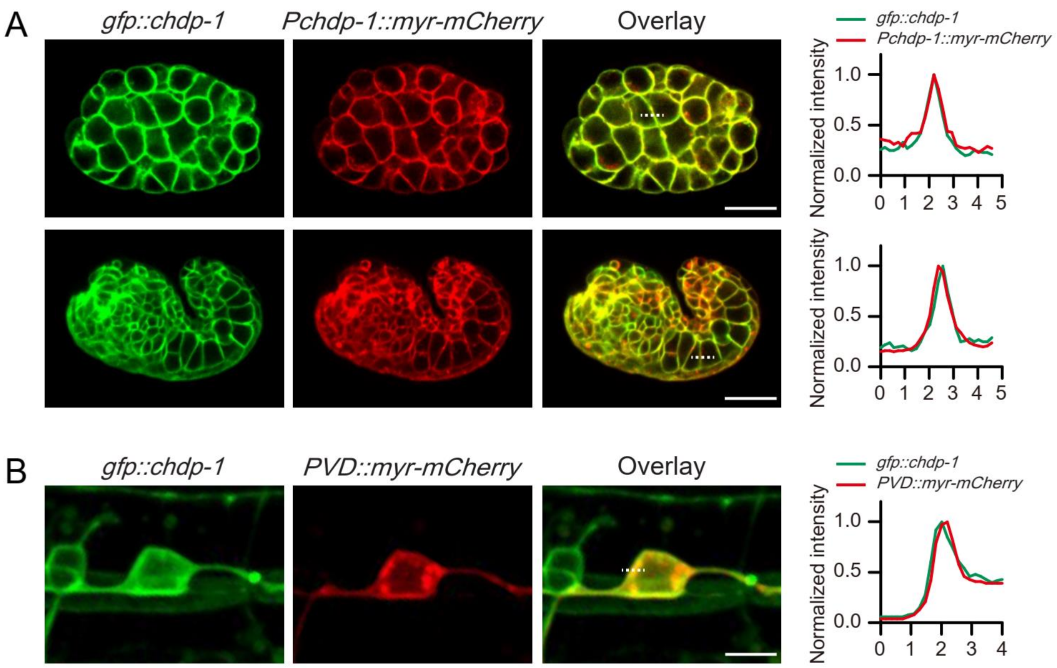

S5 Fig. Co-labeling of the endogenous CHDP-1 and the myr-mCherry reporter

2. Figure S3 should be included in the formal text as domain-function analysis is important for understanding the molecular functions of CHDP-1. Line 192-193: CHDP-1 lacking the CH domain shows punctate signals in the cytosol and CHDP-1 without the helix domain shows diffuse cytosolic distribution. These changes should be explicitly described and discussed in the Results.

We thank this reviewer for this suggestion. This figure is now included in the formal text as Fig 3. We also described and discussed the CHDP-1 lacking the CH domain and the helix domain:

However, possibly due to improper folding/ trafficking, GFP::CHDP-1 delta CH and GFP::CHDP-1 delta helix failed to localizes to the cell cortex, and displayed punctate signals and diffused cytosolic distribution, respectively (Fig 3D), consistent with the notion that the cell cortex localization of CHDP-1 is critical for its function in dendrite development.

3. Line 149: The coverage index is a bit misleading, as it quantifies the length of the anterior primary dendrite, a phenotype that is distinct from that of the higher order dendrites described in

Figures 1 and 2. It will be better to quantify the number of higher order dendrites in adulthood during the course of aging, as this is more coherent with the developmental phenotypes of the chdp-1 mutant. One could actually see that even in the wild type, the peripheral PVD dendrites deteriorate during aging, consistent with a prior report (Lezi et a., Neuron 2018).

We thank the reviewer for this suggestion. We have quantified the number of 2° and 4° branches, and found that the number of 2° was decreased for 7-day-old and 9-day-old *chdp-1* mutant animals (compared to 1-day-old adult animals, S3 Fig).

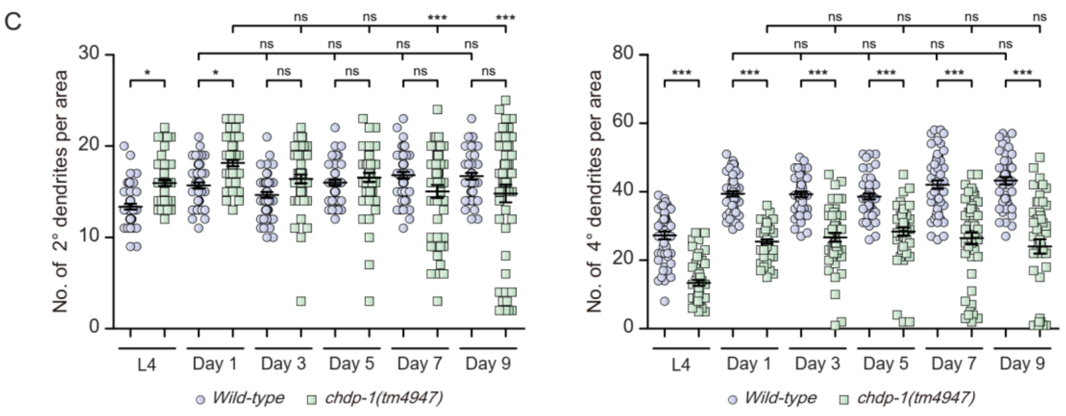

Part of S3 Fig: quantifications of 2° and 4° branches during aging

4.1. Line 202-203: The authors may want to briefly explain how NATF works to detect the endogenous expression pattern of CHDP-1.

We thank this reviewer for this suggestion. We have now added the following sentences:

Briefly, we inserted the seven copies of sequences encoding the GFP11 into the N-terminus of the endogenous CHDP-1 using CRIPSR/Cas9-mediated genome editing, and over-expressed GFP1-10 specifically in the PVD neurons using the *ser2prom3* promoter from an extrachromosomal array.

4.2. In Figure 5B, the fluorescent signals of GFP::CHDP-1 are diffuse in the PVD dendrites. One cannot conclude that CHDP-1 and moesinABD colocalize on the basis of such diffusely distributed signals, and I do not think this supports the statement that CHDP-1 is enriched in the growth cone.

We thank this reviewer for this suggestion. We have deleted the description that CHDP-1 and moesinABD colocalize. To test whether CHDP-1 is enriched in the growth cones of 2º branches, we quantified the intensity of 2º growth cone and normalized it with that of the primary dendrites. The quantification supports that CHDP-1 is enriched in the 2º growth cone (Fig 4B-4D). We also quantified the intensity of CHDP-1 in the growth cones of the primary dendrites, and normalized it to the intensity of the proximal primary dendrites. Our quantification also supports that CHDP-1 is enriched in the growth cones of the primary dendrites (Data 1).

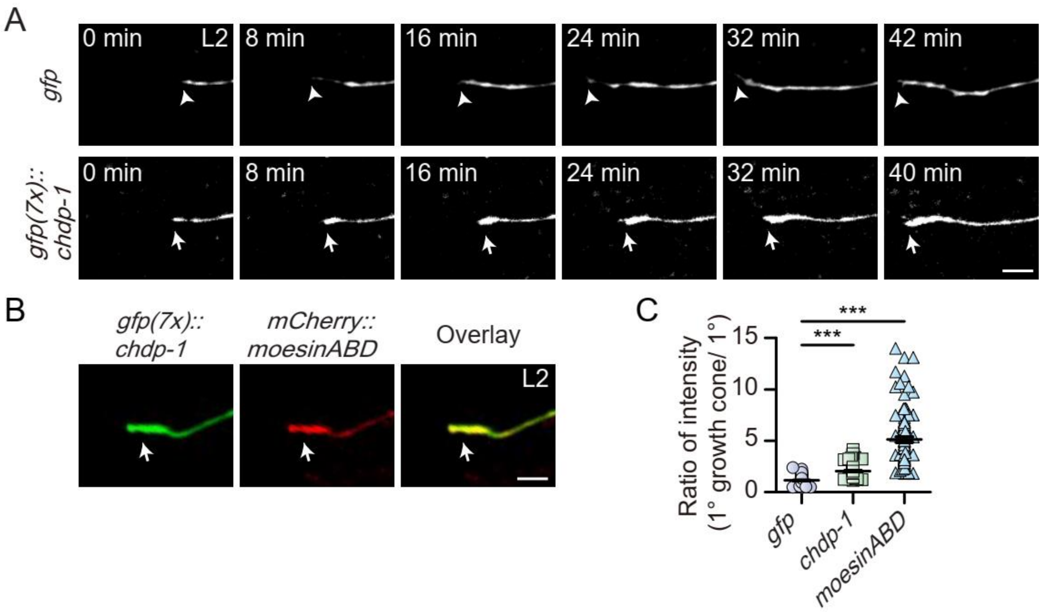

Data 1. Endogenous CHDP-1 is enriched in the 1º growth cones

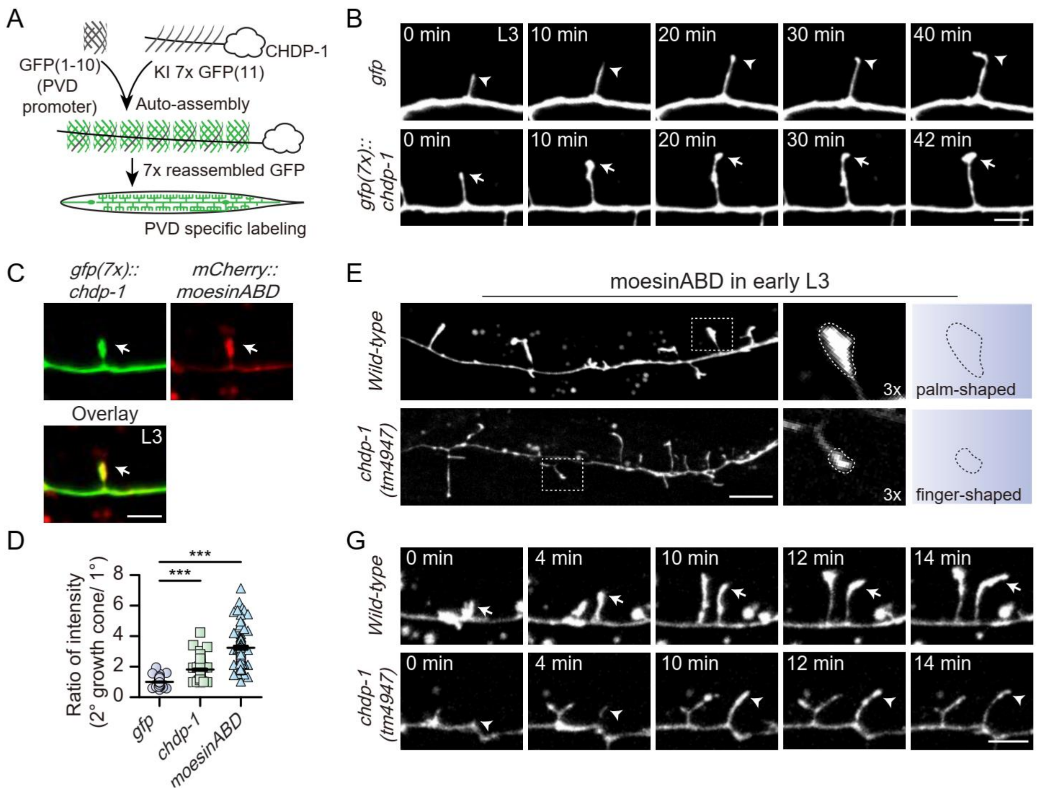

Part of Fig 4: Endogenous CHDP-1 is enriched in the 2º growth cones

4.3. The seven consecutive GFP(11) modules attached to the CHDP-1 protein could be a source of technical concern. The authors should address this issue with alternative methods.

We thank this reviewer for this suggestion. We quantified the number of branches in the *gfp(7x)::chdp-1* strain and found that there is no defect for dendrite development. These results suggest that attaching the seven consecutive gfp11 modules to the CHDP-1 protein does not affect its function in dendrite development (please see S4 Fig).

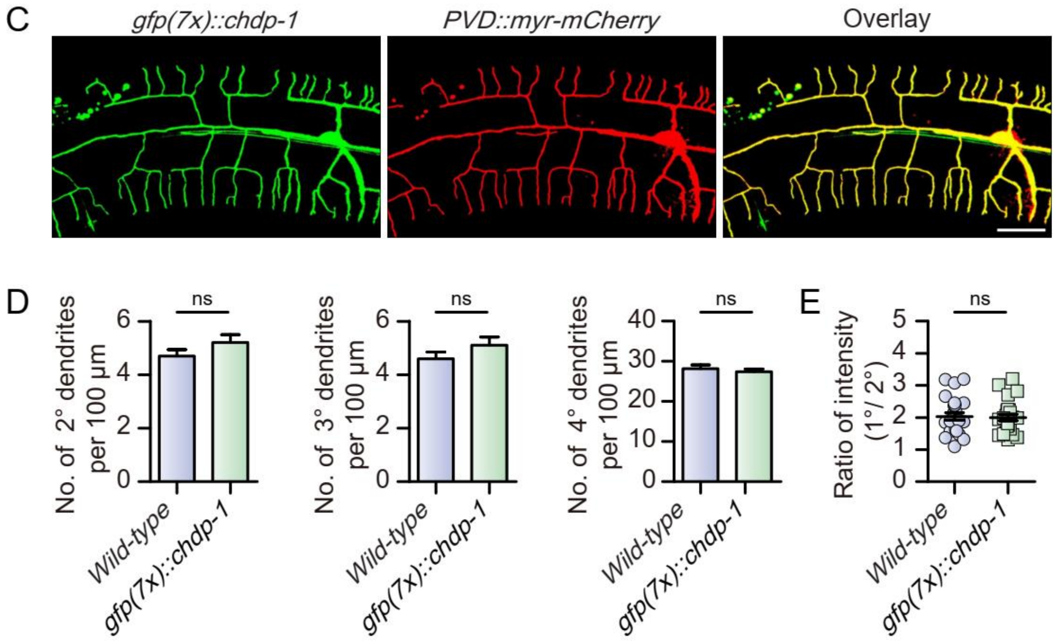

Part of S4 Fig: PVD dendrite development is normal in *gfp(7x)::chdp-1* animals

5. Figure 5D and 5F: It seems to the reviewer that at L3, the dendrite growth cones in the wild type display extended palm-shaped morphology and undergo dynamic changes in their appearance, whereas those in the chdp-1 mutants are thin, pointed and less dynamic. The current quantification of the growth cone area does not highlight the dynamic nature of the growth cone morphology. Did the authors quantify the same 100 growth cones in respective genotypes? This is hard to imagine, as not all growth cones are present at any given time points during the recording: some are yet to appear while others are retracting. The authors need to provide more detailed account of their time-lapse imaging methods and characterize the dynamic nature of the dendrite growth cones.

We thank this reviewer for this suggestion. We did not quantify the same 100 growth cones. We have quantified the percentage of the palm-shaped and finger-shaped growth cones. The results are showed in Fig 4E-4F. We also added the following sentences to describe these results:

In the wild-type animals, more than 80% of the moesinABD-labeled growth cones of the 2° branches showed palm-like shape. In contrast, nearly all the growth cones of the 2° branches in the *chdp-1(tm4947)* animals showed finger-like shape (Fig 4E-4G).

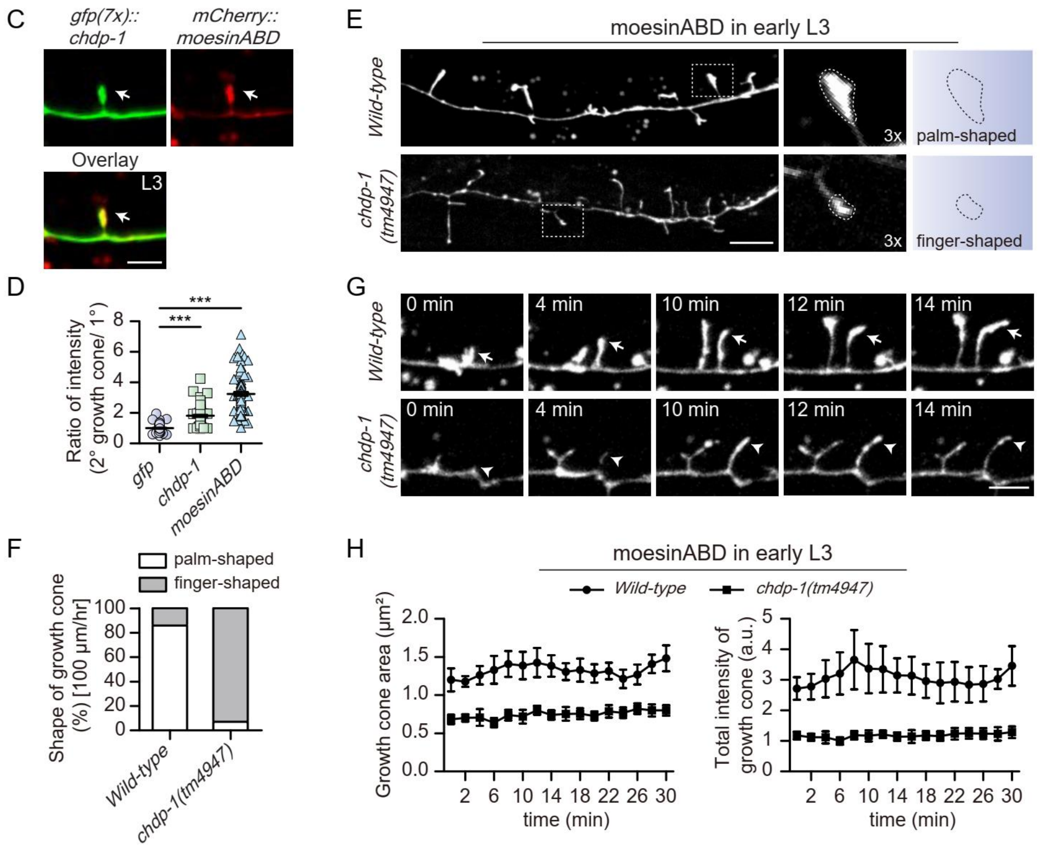

Part of Fig 4: Morphologies of 2° growth cones in WT and *chdp-1* mutants

6.1. Line 235-238: The authors imply that NLP-12 is secreted from the peripheral PVD dendrites, which is quite unusual. They should also examine whether NLP-12::Venus in the PVD axon is also altered in the chdp-1 mutant.

We thank this reviewer for this suggestion. A previous study from Dr. Kang Shen's lab demonstrated that NLP-12 is largely secreted from the dendrites but not the axons (Li Tao et al., 2019, Dev Cell). Please see below:

Next, we asked where in the PVD is NLP-12 secreted. NLP-12::Venus is enriched in the PVD axon but also localized to the dendrites (Figure 6A). To test whether NLP-12 is released from the axon or dendrites, we pursued two strategies. First, we examined the localization of NLP-12 in the *unc-104* PVD-specific RNAi worms. This manipulation almost eliminated the axonal NLP-12 in PVD without affecting dendritic NLP-12 puncta (Figures S7G and S7H). Consistent with the dendritic secretion model, the NLP-12::Venus fluorescence in the coelomocytes is unchanged from WT controls, suggesting that dendritic secretion likely accounts for much of the secreted NLP-12 (Figure S7I). These data suggest that NLP-12 is unlikely to be released by the axon and implicate a local dendritic release mechanism.

So, we mainly focused on the release of NLP-12 from the PVD dendrites in WT and *chdp-1* mutants in this study.

6.2. NLP-12::Venus puncta in the peripheral PVD dendrites are sparse (Figure 7A, 7B, 7D and 7E), with a mean density of one puncta per 100 um of dendrite length. This seems to be a pretty low number for something that is so important for proprioception and neuromuscular function.

We thank this reviewer for this comment. We think this is likely due to the dynamic and efficient transport and secretion.

6.3. Figure 7C and 7D were poorly explained in the text. Please explain the design of the GBP assay and the interpretation of the data.

We thank this reviewer for this suggestion. We have added the following sentences to explain the design of the GBP assay:

Briefly, GFP nanobody, which is also known as the GFP binding protein (GBP), was fused at the N-terminus of the single transmembrane domain protein SAX-7 and the fused protein was specifically expressed in the epidermal cells using the *Pdpy-7* promoter. SAX-7 formed stripes to guide the formation of 3° and 4° branches of the PVD neurons. Thus, once NLP-12::Venus was secreted from these branches, the neuropeptide was captured by the locally expressed GBP::SAX-7 due to the high binding affinity between GBP and the Venus protein.

6.4. In Figure 7D, does the “wild type” animal also express Pdpy-7::GBP::SAX-7, as in the sax-7 and chdp-1 mutants? The label of Pdpy-7::GBP::SAX-7 is inconsistent between 7D and 7E.

We thank this reviewer for pointing this out. The incorrect labeling is now corrected.

7. Line 243-244: To show that NLP-12 dendritic transport is disrupted in the chdp-1 mutant, the authors should quantify NLP-12::Venus puncta in the primary PVD dendrite and/or measure NLP-12::Venus movement by kymograph analysis.

We thank this reviewer for this suggestion. We have quantified the NLP-12::Venus in the primary PVD dendrites (please see S8 Fig).

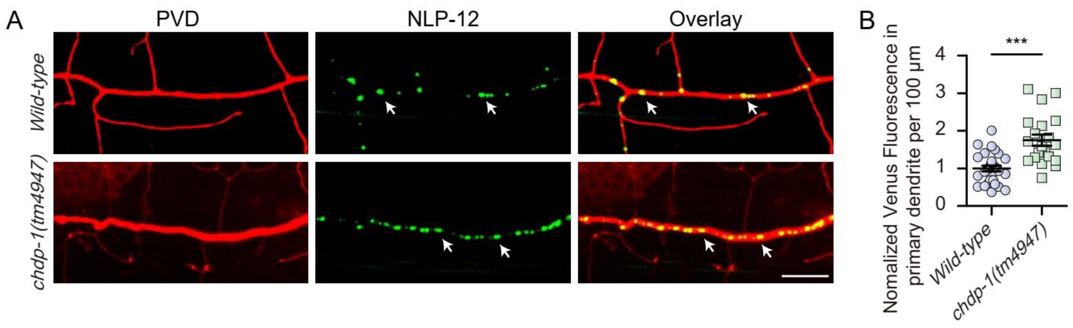

S8 Fig: Accumulation of dense-core vesicles in the 1° dendrites of *chdp-1* mutants

8. It is unclear how CHDP-1, which the authors claim to be involved in the assembly of cortical actin near the cell membrane, is required for vesicle transport and trafficking, as actin species for this transport function is distinct from that underneath the cell cortex. The authors should offer some speculation.

We thank this reviewer for this suggestion. We think that the vesicle transport and trafficking defects in the chdp-1 mutants are due to abnormal microtubule assembly in the high-order branches. Please see the following paragraph:

In the high-ordered branches, loss of *chdp-1* reduced not only the cortical actin assembly, but also the microtubule assembly. Two models can explain the decreased microtubule cytoskeleton. (1) CHDP-1 directly acts as a microtubule assembly factor. (2) The effect of CHDP-1 on microtubule

assembly is secondary of its function in cortical actin assembly. Actin cytoskeleton often interacts with microtubule cytoskeleton [1,47]. Numerous proteins have been reported to mediate structural interactions between microtubules and actin, such as coronin, and Drebrin-EB3 [48,49]. Disrupting of actin cytoskeleton can lead to microtubule assembly defects [50]. Thus, it is conceivable to hypothesize that the cortical actin in the high-ordered branches can serve as a platform to promote microtubule assembly and stabilization. Future studies are needed to tell which model is correct.

9. Figure 8: The data showed that both plus end-out and minus end-out microtubules (MTs) exist in the secondary PVD dendrites, based on the EBP-2 comet patterns. In the chdp-1 mutant, all EBP-2 comets were absent, raising an intriguing possibility that CHDP-1 is required for the assembly of both plus end-out and minus end-out MTs. Given that CHDP-1 is a cell cortex protein, how does it regulate the assembly of MTs, especially for MTs of opposite polarity? Although in Discussion (Line 343-344), the authors favored the model that CHDP-1 regulates MTs indirectly via actin assembly, there is no evidence for this. The authors may want to explore whether CHDP-1 is associated with the microtubule cytoskeleton.

We thank this reviewer for this suggestion. We have generated a strain to simultaneously examine the subcellular localization of CHDP-1 and TBA-1. It seems that some MTs are co-localized with CHDP-1 near the cell cortex (Data 2). Thus, it is possible that CHDP-1 directly associates with MTs and regulates MT assembly. We also discussed about this possibility in the discussion.

In the high-ordered branches, loss of *chdp-1* reduced not only the cortical actin assembly, but also the microtubule assembly. Two models can explain the decreased microtubule cytoskeleton. (1) CHDP-1 directly acts as a microtubule assembly factor. (2) The effect of CHDP-1 on microtubule assembly is secondary of its function in cortical actin assembly. Actin cytoskeleton often interacts with microtubule cytoskeleton [1,47]. Numerous proteins have been reported to mediate structural interactions between microtubules and actin, such as coronin, and Drebrin-EB3 [48,49]. Disrupting of actin cytoskeleton can lead to microtubule assembly defects [50]. Thus, it is conceivable to hypothesize that the cortical actin in the high-ordered branches can serve as a platform to promote microtubule assembly and stabilization. Future studies are needed to tell which model is correct.

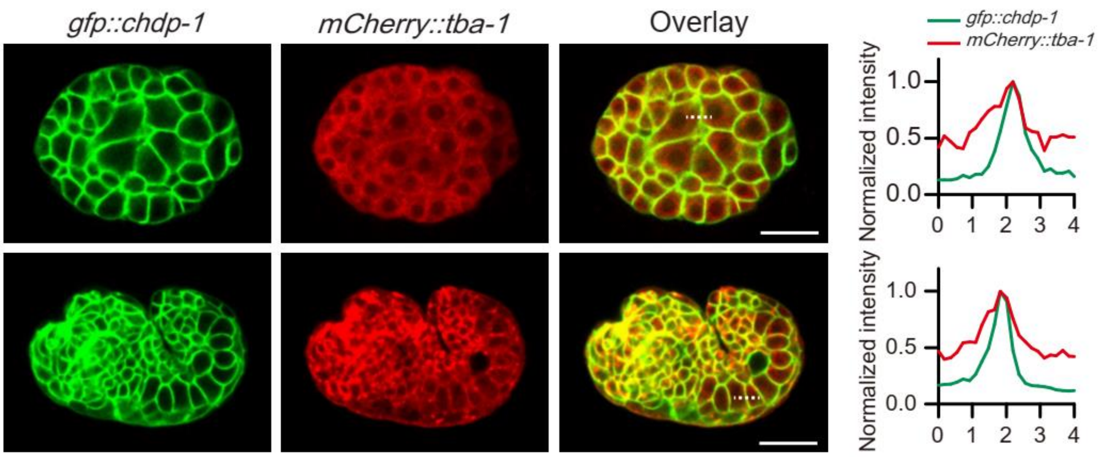

Data 2: Co-labeling of CHDP-1 and TBA-1 in embryos.

Minor comments:

1. The Materials and Methods section should be significantly substantiated. Many experiments are inadequately explained, preventing the readers to grab essential details of the experiments. This is particularly evident with the split-GFP assay for cell-specific detection of CHDP-1 expression and the local neuropeptide secretion assays.

We thank this reviewer for this suggestion. We have added more information in the materials and methods section. Especially, we have added two paragraphs to describe how we performed the split-GFP assay for cell-specific detection of CHDP-1 expression and the local neuropeptide secretion assays.

Split-GFP Assay for Cell-Specific Detection of CHDP-1 Expression

This assay was perfomed as described by Siwei He *et al*. Briefly, the coding sequence for $GFP11^{7x}$ was amplified from a previously published plasmid (Addgene #70224). The PCR product was assembled with two homology arms (~ 500 bp each) amplified from the N2 genomic DNA, and the digested pSM delta plasmid as the backbone via the Gibson assembly protocol to generate the repair template plasmid pZT105. Similar CRISPR knock-in strategy was used as how *gfp::chdp-1* was generated, except that pZT105 was used in this experiment. Successful knock-in animals were obtained through PCR-based genotyping, and no extra mutation was found by Sanger sequencing.

The coding sequence for GFP1-10 was amplified from a previously published plasmid (Addgene # 70219). The PCR product was cloned into a plasmid in which the PVD-specific promoter ser2prom3 was previously inserted. This plasmid (pZT106, 20 ng/μl), *Podr-1::rfp* (50 ng/μl) and *Pmyo-2::mcherry* (2 ng/μl) were injected into the *zac283[gfp11(7x)::chdp-1]* strain. Stable transgenic lines were isolated via the expression of the co-injection markers and subjected to confocal imaging.

Local Neuropeptide Secretion Assay

This assay was performed as described by Li Tao et al. Briefly, pZT143 *ser2rom3::nlp-12::venus::sl2::mcherry* (20 ng/μl), pLT110 *Pdpy-7::gbp::sax-7* (10 ng/μl, kindly provided by Dr. Kang Shen), *Pmyo-2::mcherry* (2 ng/μl) and *Podr-1::rfp* (50 ng/μl) were injected into *sax-7(nj48)* or *chdp-1 (tm4947)* mutant animals. Stable transgenic lines were obtained and subjected to confocal imaging.

2. Figure 1D: What is the orientation of the STED images of secondary dendrites here?

We thank this reviewer for pointing this out. We have added information for the orientation of the STED images of 2° branches in the figure legends of the revised S1 Fig (left: close to the 1° dendrite. Right: close to the 3° branches).

3. The genotype label chdp-1(tm4947) in all the figures should be italicized. Please carefully check this.

We thank this reviewer for this suggestion. Corrected.

4. Line 129: Change chdp-1-/- to chdp-1(tm4947) throughout the paper, and indicate that tm4947 is a representative null, deletion allele where it first appears in the manuscript.

We thank this reviewer for this suggestion. All changed.

5. Line 130-133, 137-140: There is no need to describe the quantitative results in numerical

details, as these could be read from the respective figures directly. Please simplify tis and remove redundant descriptions throughout the paper.

We thank this reviewer for this suggestion. All removed.

6. Figure 2A and 2F: Please show snapshots of time-lapse imaging that have both growing and retracting secondary dendrites. There are only growing dendrites in the current figure.

We thank this reviewer for this suggestion. Done (please see Fig 1D and S2 Fig).

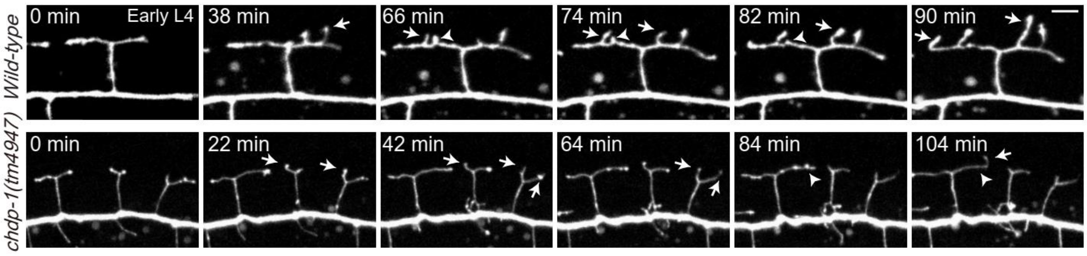

Fig 1D: Outgrowth and retraction of 4º branches in WT and *chdp-1* mutants

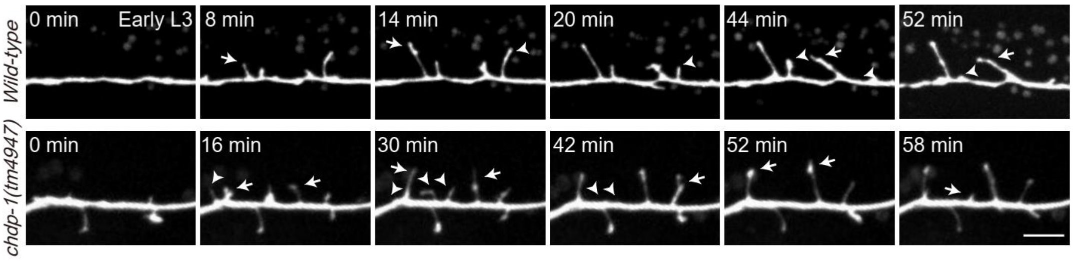

Part of S2 Fig: Outgrowth and retraction of 2º branches in WT and *chdp-1* mutants

7. Figure 6: The dendrite defects of the chdp-1 mutant are "relatively mild" compared to those in the dma-1 mutant. However, both display similar proprioceptive defects quantified by the amplitude and wavelength of the sinusoidal movements on the agar. The authors should offer some explanation.

We thank this reviewer for this suggestion. We have added one sentence to explain it.

These results reveal that although the high-ordered branches could be generated when *chdp-1* is mutated, they are non-functional for proprioception.

8. Line 234: Please change "why" to "how". As scientists, we deal with HOW things happen rather than WHY they happen.

We thank this reviewer for this suggestion. Changed.

9. Line 247: It is endoplasmic "reticulum", not "reticulon".

We thank this reviewer for this suggestion. Changed.

10. Figure 7F: The signal of ER labeled by SP12 is continuous and diffuse in the primary, secondary and some of the tertiary PVD dendrites. I would have expected a puncta appearance, as other subcellular compartments in the neuronal process. Could the authors explain this peculiar pattern of dendritic ER or confirm it with another ER marker such as KDEL?

We thank this reviewer for pointing this out. The pattern we observed is consistent with two previous findings (Melissa Rolls et al., 2002, MBoC; Xianzhuang Liu et al., 2019, Nat Commun). Please see below:

To visualize the ER in *C. elegans* neurons, FP-SP12 was expressed under control of the *glr-1* promoter (Figure 3A). The *glr-1* promoter drives expression in different classes of motorneurons and interneurons (Hart *et al.*, 1995), quite a few of which send neurites into the ventral nerve cord. Most of the cell bodies are located in the ganglia near the nerve ring, although a few are in the retrovesicular ganglion posterior to the nerve ring, and some are in the tail ganglia. FP-SP12 fluorescence was visible continuously in the ventral nerve cord (Figure 3A, labeled V), indicating that the neurites contain ER. In addition to neuronal expression, the vectors with the *glr-1* promoter gave some expression in several head muscles (see Figure 1B).

To determine whether any difference in localization of RER and general ER membrane proteins could be detected in neurons, FP-fusions to the two classes of predicted pro-

RER Membrane Protein Targeting in Neurons

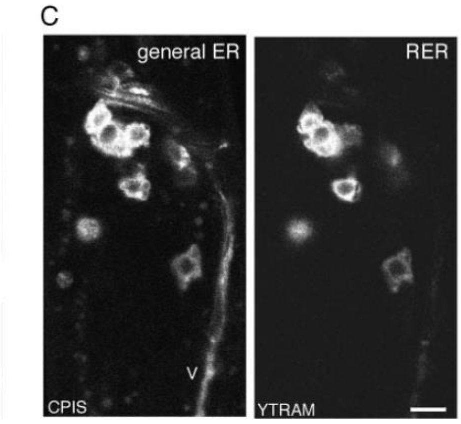

NATURE COMMUNICATIONS | https://doi.org/10.1038/s41467-019-08478-6 ARTICLE

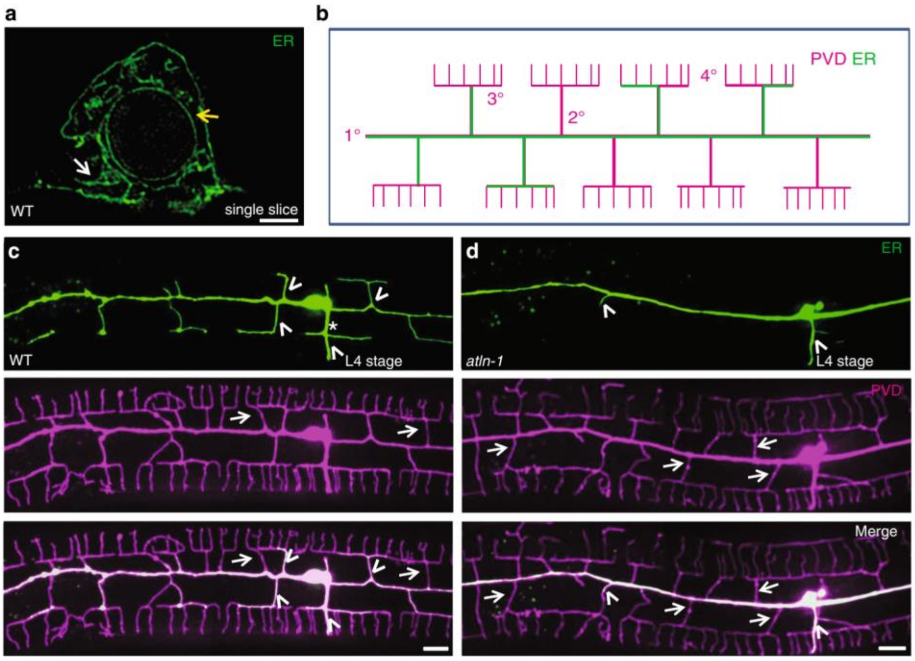

11. Figure S5A: There seems to be a diffuse background GFP signal in addition to the more punctate DMA-1::GFP. It is not easy to appreciate the presence of the punctate signals. The authors need to provide their criteria of quantifying DMA-1::GFP vesicles. Please explain the exoc-8 genetic background in the main text.

We thank this reviewer for this suggestion. If there is a DMA-1::GFP vesicle that is at least 2-fold brighter than the diffuse background GFP signal, it is counted as a vesicle. This information has been added into the related figure legends. We have also explained the *exoc-8* genetic background in the main text.

In the wild-type animals, a small number of DMA-1::GFP positive vesicles could be observed in the high-ordered branches. However, it was extremely difficult for us to identify any vesicles in the high-ordered branches. DMA-1::GFP strongly labeled the high-ordered branches in both the wild-type and *chdp-1(tm4947)* mutant animals, which makes it a bit difficult to quantify the number of vesicles. We took advantage of the *exoc-8* mutants, in which the docking/ fusion of DMA-1 vesicles onto the dendritic membrane was strongly affected and confirmed that CHDP-1 was indeed required for the transport of the secretory vesicles/ endosomes from the primary dendrites into the high-ordered branches (S10 Fig).

12. Line 303: The statement that SAX-1 is a negative regulator of actin assembly is based on its antagonism of CHDP-1. However, direct evidence in support of this conclusion is missing in the current study. The authors are suggested to tone it down or clearly indicate that this is a speculation.

We thank this reviewer for this suggestion. We have clearly indicated that this is a speculation.

Together, our results suggest that SAX-1 acts opposingly to CHDP-1, and we speculated that it is a negative regulator of cortical actin assembly.

Reviewer #2: The manuscript by Zhao et al. describes the role for the calponin homology containing protein, CHDP-1 in dendritic morphogenesis of the nociceptive receptor neuron, PVD in C. elegans. CHDP-1 was previously shown by the authors to be associated with the CED-10/RAC-1 to regulate actin cytoskeleton in BDU & PLM neuron. In this study, they extend the functional analysis of CHDP-1 to PVD morphogenesis. Through a set of experiments, they show

that CHDP-1 function is required for proper dendritic arborization. CHDP-1 function in the PVD to modulate actin assembly, distribution of organelles and microtubules and proprioception. The authors suggest that CHDP-1 regulates actin assembly which in turn affects the intracellular cytoskeleton and that this is necessary for the development, maintenance, and function of the PVD dendrites. This an interesting paper with several well executed experiments and adds a new player into the mix of components that is required for the dendritic patterning in the PVD.

We thank this reviewer for the positive comments on our manuscript.

In general, I found the paper to be very dense with a lot of different experiments testing various hypotheses. Some of the data are strongly supported by the evidence provided by the authors however a few I find to be preliminary. It would be useful if the authors could distil the information in the main figures by only including the data that shows the strongest evidence. The main takeaway for me is that chdp-1 is involved in proper organization of the cytoskeleton and organelles in the higher order branches. This is a cell autonomous function and that this requires the CH and the helix within chdp-1. Lack of chdp-1is leads to dendrite degeneration and defective proprioception due to impaired branches. This function may not be through the ced-10 protein but is suppressed by the sax-1 kinase which can affect the actin protrusion dynamics arguing that chdp-1 is affecting an actin dependent process.

We thank this reviewer for this excellent suggestion. We have distilled the information in the main figures by only including the data that shows the strongest evidence. Briefly, we have combined our previous Fig 1 and Fig 2 to form the new Fig 1. The STED results are now showed as part of S1 Fig. The previous Fig 3 is now showed as the S3 Fig. We have also combined our previous Fig 7 and Fig 8 to form the new Fig 6 (the results for ER and mitochondria transport are now showed in S9 Fig). We have also combined our previous Fig 9 and Fig 10 to form the new Fig 7, and the PVD conditional knock-out of *sax-1* and the proprioception phenotype in the *chdp-1; sax-1* double mutants are now showed in S13 Fig.

Specific comments.

1. Results for chdp-1 mutant effects on 2º branches are confusing. Figure 1B shows an increase of 2º branches in mutants of chdp-1 vs. WT. Representative images (Figure 1A), however, appear to show similar 2º branches in all three panels. It is that clear chdp-1 mutants affect the 2º, 3º & 4º branching dynamics/frequency based on the time lapse data. The strongest phenotype lies in the branching defects of higher order dendrites. Given the penetrance of that phenotype it, focusing on the 3º & 4º branching defects alone would be sufficient to do the functional analysis done with the CHDP-1 protein. Also, Figures 1D, E & F which shows the width of the primary dendrite is not adding much as it is difficult to know the exact parameters that is being measured as well as the reason for swelled 1º dendrite.

We thank this reviewer for this suggestion. We apologize to have not shown the most representative images for WT and *chdp-1* mutants. We hope that it is now clear that *chdp-1* mutants grow more 2º branches than that of WT. Following this reviewer's suggestion, we have moved the STED imaging results into S1 Fig, and focused on the branching of 4º dendrites. The time-lapse results of 2º dendrites are now showed in S2 Fig. Since we quantified the number of 2º branches and the normalized intensity of them in multiple main figures and supplementary figures, we hope that this reviewer will allow us to keep the quantifications for 2º in Fig 1, but not move them into a supplementary figure.

2. In Fig5 the authors very nicely use a splitgfp system to visualize CHDP-1 exclusively in PVD. They also use mCherry:moesinABD as a growth cone marker and states that "GFP7x::CHDP-1 was enriched in the dendritic growth cones, and colocalized with the F-actin 205 probe mCherry::moesin actin binding domain (moesinABD)". I am struggling to see the enrichment of CHDP-1 in the growth cones. From the images in Fig.5A it does look like the CHDP-1 is uniform. Would it be possible to use a different counter marker such as membrane to support that CHDP-1 is indeed enriched in growth cone? An alternate possibility is to look at the growth cones of 1º dendrite which are much larger in size and is also much more defined.

We thank this reviewer for this suggestion. We have measured the intensity of GFP(7x)::CHDP-1 in the growth cones of both the primary dendrites and secondary branches. CHDP-1 is indeed enriched in the growth cones as indicated by the quantifications. Please see Fig 4B-4D and Data 1.

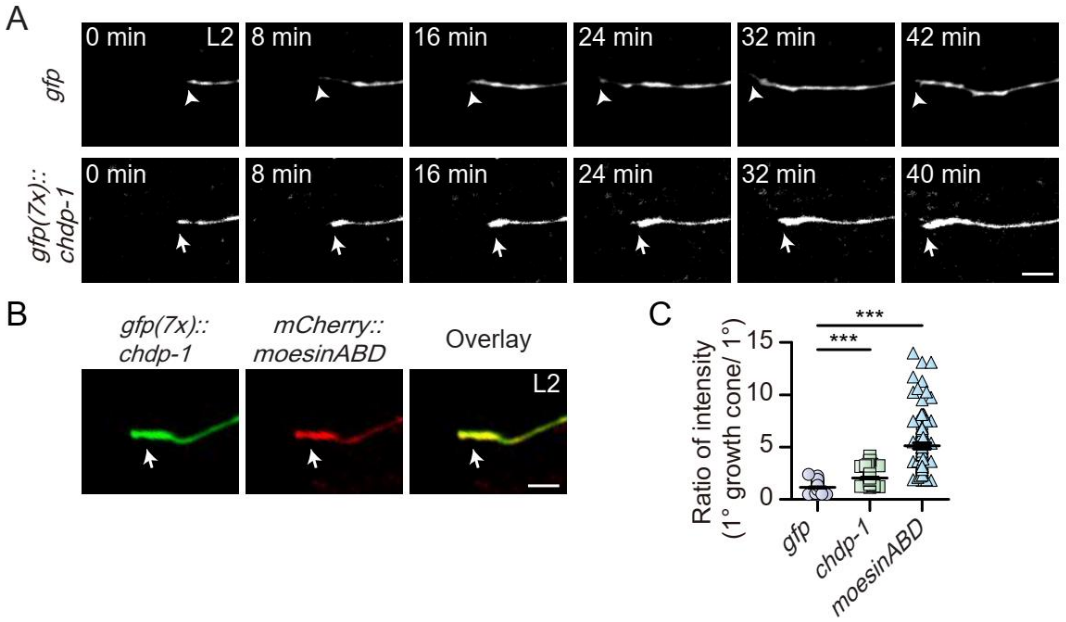

Data 1. Endogenous CHDP-1 is enriched in the growth cones of the 1º dendrites

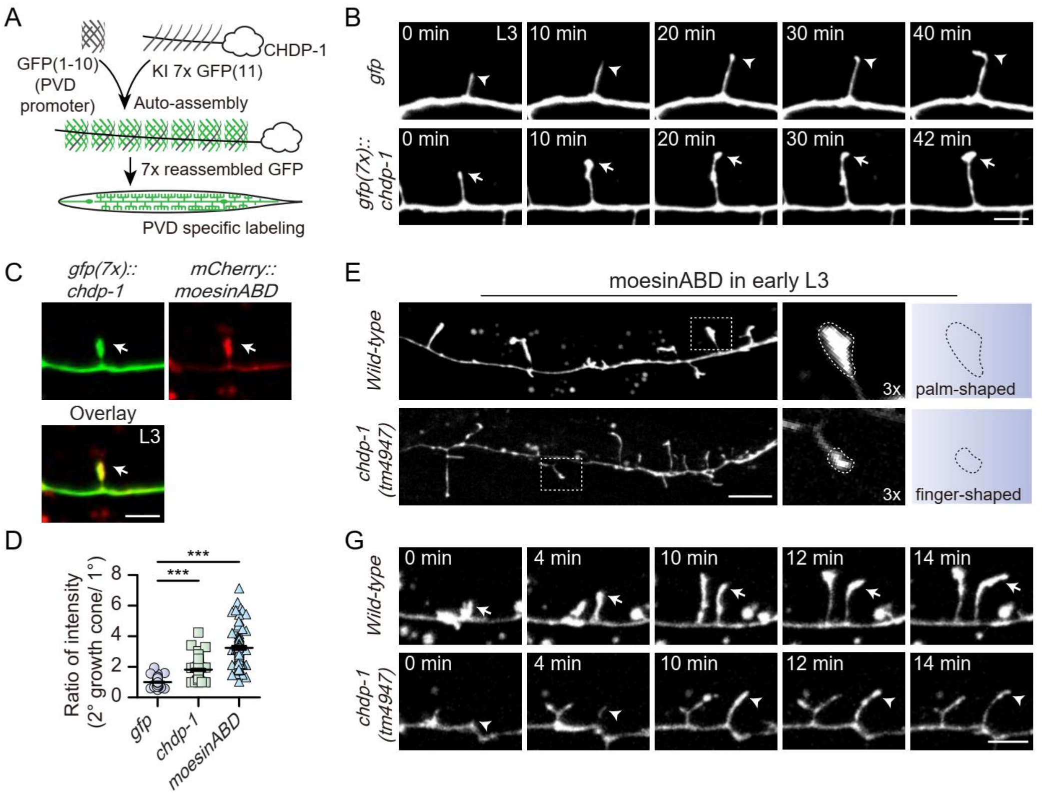

Part of Fig 4: Endogenous CHDP-1 is enriched in the 2º growth cones

Minor comments/suggestion

Figures 1 & 2 could be combined and some of the data from figure 1 (STED & 2º branch quantification) could be moved to supplemental data section. The data in on higher order branching and the time lapses are clearly strong to support chdp-1 mutant phenotype. Also, it might be worth combining the dendritic regeneration and proprioception data as it is a functional consequence to the neuron.

We thank this reviewer for this suggestion. We have combined the old Figures 1 and 2, and moved the STED results into S1 Fig. We have also moved the dendritic degeneration results into S3 Fig. Since we quantified the number of 2º branches and the normalized intensity of them in multiple main figures and supplementary figures, we hope that this reviewer will allow us to keep the quantifications for 2º in Fig 1, but not move them into a supplementary figure.

Reviewer #3: The manuscript by Zhao et al describes the function of CHDP-1 in PVD neuron development. The mutant shows some interesting phenotype: there are ectopic 2o and 3o dendrites, however, less 4o dendrite. The 4o dendrites are not abolished though like other F-actin cytoskeleton related mutants such as hpo-30, tiam-1, act-4, dma-1. The phenotype seems to be more specific to ectopic 2o branches emerging from the primary one. Although it is an interesting study and it can be published in PLOS-GENETICS. However, several questions remain in terms of how CHDP-1 regulates the cytoskeleton element in the PVD neuron.

We than this reviewer for the positive comments on our manuscript.

1) It is not clear how they conclude CHDP-1 affects cortical actin cytoskeleton specifically. It may affect cytosolic F-actin structures as well. It seems in general F-actin cytoskeleton is dim in the mutant dendrites.

We than this reviewer for pointing this out. In a previous study, we reported that GFP::CHDP-1 expressed from a transgene co-localizes with the plasma membrane marker myr-mCherry in the

cell bodies of PLM neurons (Guan et al., 2016, Plos Genet). To determine whether the endogenous CHDP-1 also localizes to the cell cortex, we co-expressed myr-mCherry in this strain. As the results showed in S5 Fig, these two proteins display co-localization. These data suggest that CHDP-1 is mainly a cell cortex-localized protein. Thus, CHDP-1 may mainly regulate actin assembly close to the cell cortex. We do agree with this reviewer that loss of *chdp-1* may affect cytosolic F-actin structures as well. There might be a small pool of CHDP-1 proteins which are not localized onto the cell cortex.

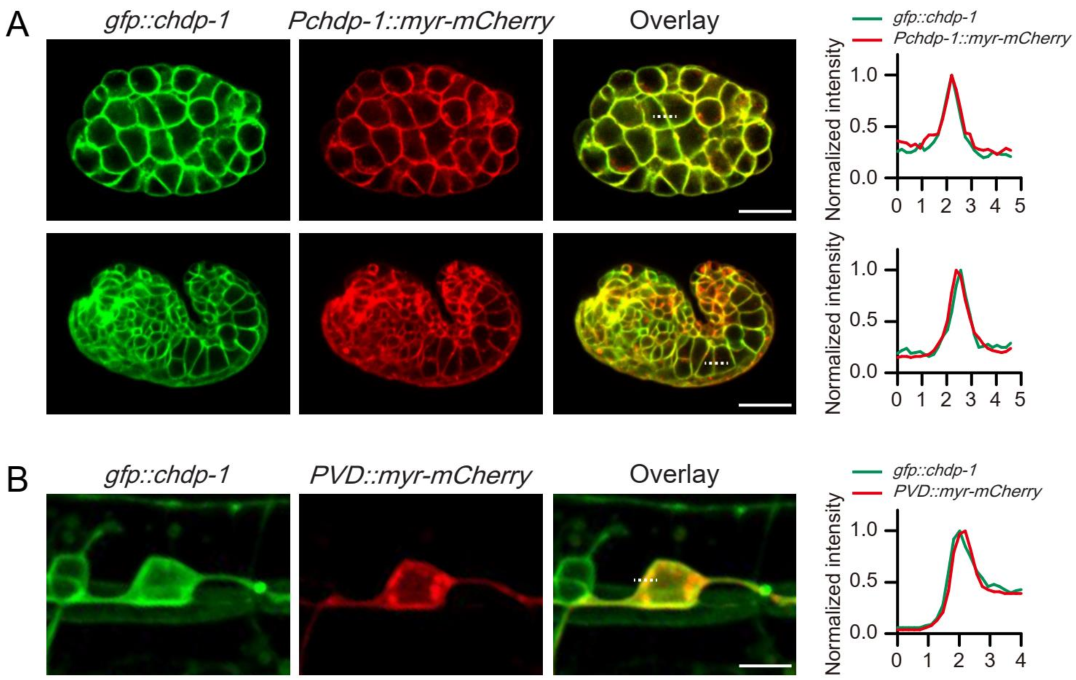

S5 Fig. Endogenous CHDP-1 localizes to the cell cortex

2) It is not clear whether CHDP-1 promotes assembly or disassembly of F-actin. Some photo-bleaching experiment followed by recovery could tell how F-actin dynamics is affected.

We thank this reviewer for this suggestion. We observed decreased F-actin in the growth cones of 2º and 4º branches when chdp-1 is mutated. These results likely suggest that CHDP-1 promotes F-actin assembly. However, F-actin assembly and disassembly is tightly coupled. For example, increased F-actin disassembly can generate more G-actin, which can be used for F-actin assembly. Previously we reported that over-expressing CHDP-1 causes reduced actin in the stress fibers

(Guan et al., 2016, Plos Genet). So, it is possible that CHDP-1 promotes F-actin assembly or disassembly.

To explore whether CHDP-1 genetically interacts with players in actin disassembly, we over-expressed UNC-60 in both WT and *chdp-1* mutant genetic background. Interestingly, over-expression of UNC-60 partially restored the intensity of 2º and 3º branches (Data 3).

We do appreciate this reviewer for the FRAP experiments. Unfortunately, we do not have a FRAP module on our spinning disk confocal. In addition, as we discussed above, we think this experiment may still unable to tell whether CHDP-1 promotes actin assembly or disassembly. We plan to perform proximity-labeling to identify the downstream proteins which act together with CHDP-1. We hope that identification of the functional partners of CHDP-1 will answer this question in the near future.

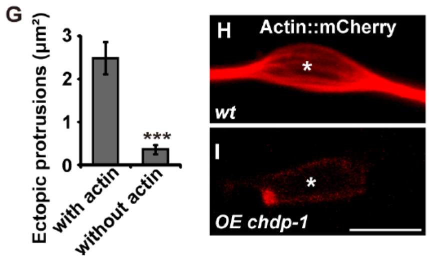

Fig 4. *chdp-1* promotes cell protrusion. (A) The PLM cell body displays a spindle-like cell shape in wild type. (B-C) Ectopic cell protrusions (arrowheads) appear on PLM cells (outlined by yellow dashed lines) when *chdp-1* is over-expressed (*OE chdp-1*). PLM is labeled by P*unc-86*::Myr::GFP. (D) Quantification of PLM cells in wild type and *chdp-1* overexpression animals with ectopic protrusions. (E) GFP::CHDP-1 co-localizes with the plasma membrane marker Myr::mCherry on ectopic protrusions. (F) Some protrusions contain actin (arrows) and some do not (arrowheads). (G) Quantification of protrusion size with or without actin. $n \geq 30$. (H-I) The stress-fiber-like structures (H) disappear in *chdp-1* overexpression animals (*OE chdp-1*) (I). On the confocal images, asterisks label the nuclei within cell bodies. Data are presented as mean ± SD; ***$p < 0.001$.

doi:10.1371/journal.pgen.1006163.g004

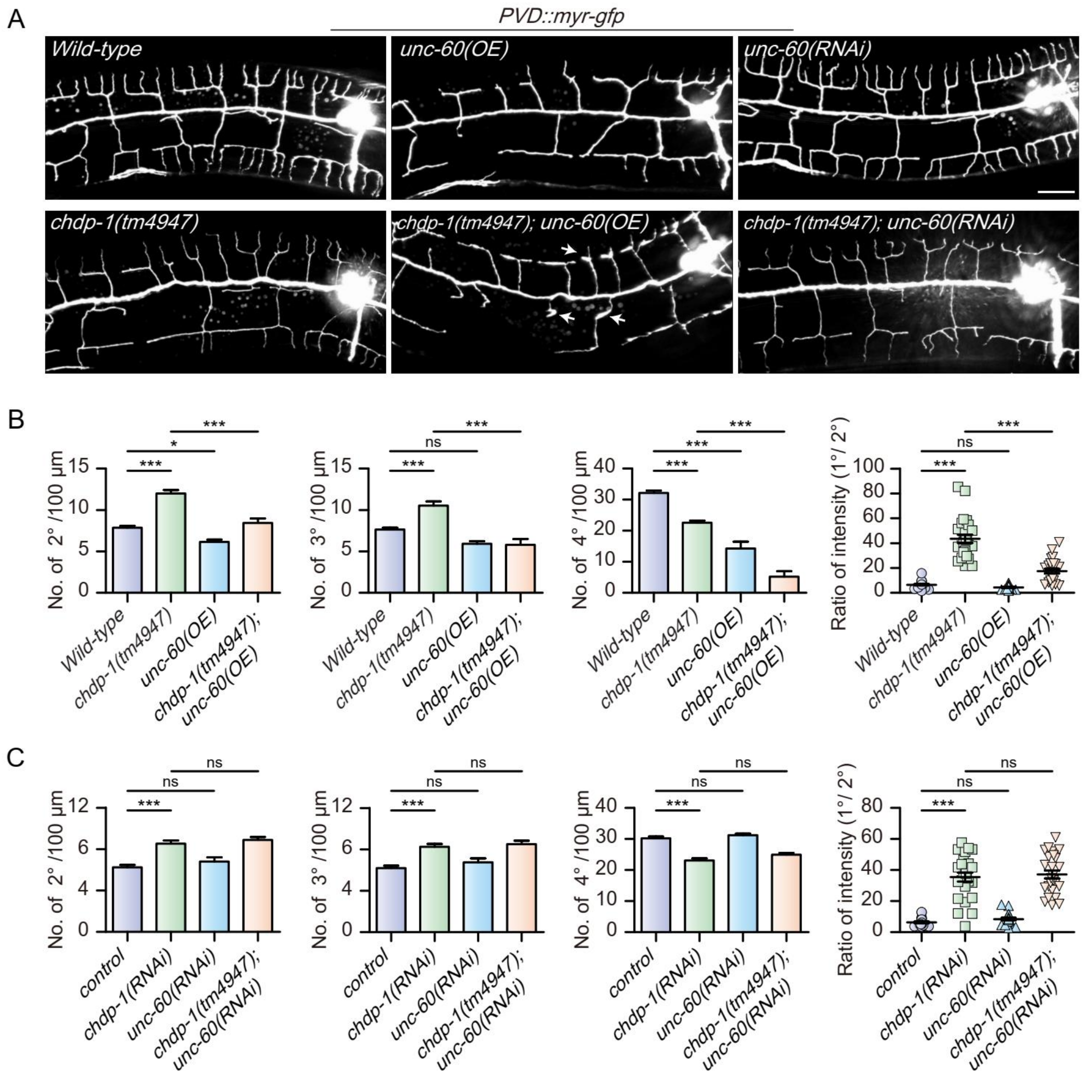

Data 3: Analyses of the genetic interaction between *chdp-1* and *unc-60*

3) Can it affect cross-linking of F-actin through non-muscle myosin? I think both drugs and mutants are available to test this

We thank this reviewer for this suggestion. We used RNAi and somatic CRIPSR to knock-down *nmy-1* and *nmy-2,* and did not see any phenotype similar to *chdp-1* mutants (Data 4). Note that a small percentage of *nmy-1* RNAi and PVD-specific conditional knock-out animals showed 3° self-avoidance defects, which is consistent with a previous published work (Lakshmi Sundararajan et al., 2019, Plos Genet).

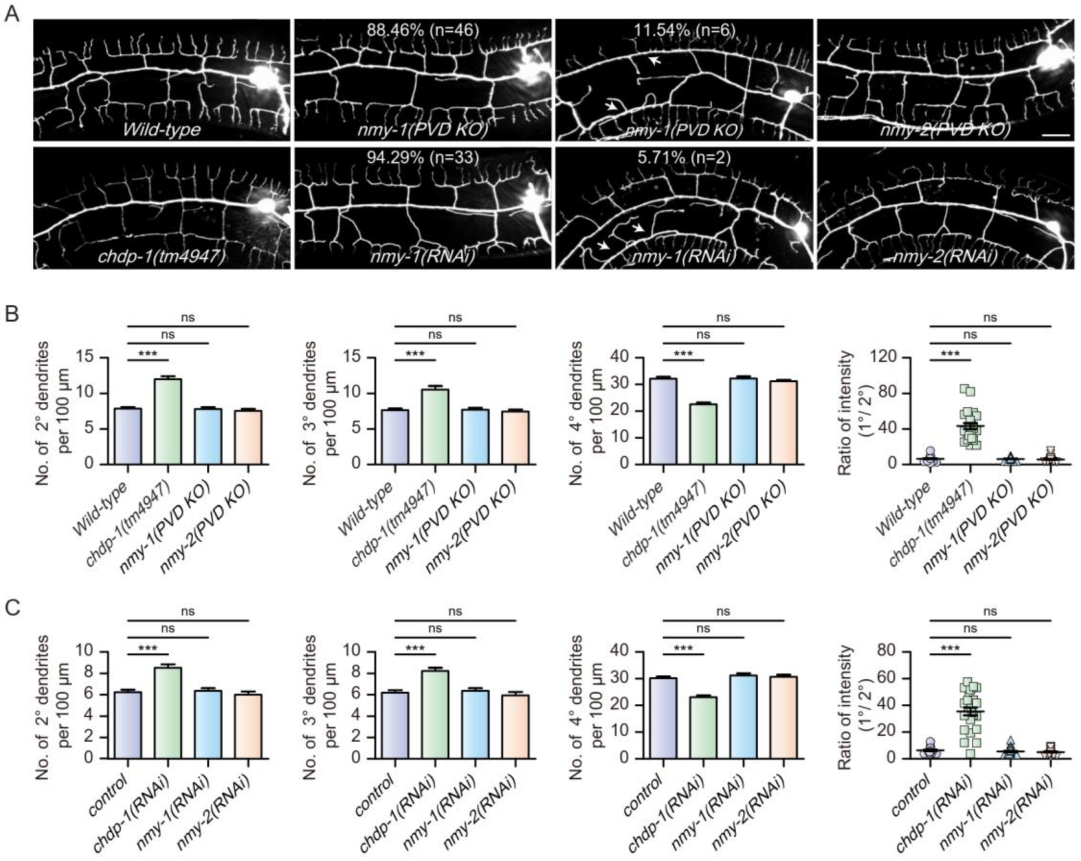

Data 4: PVD dendrite morphologies in *nmy-1* and *nmy-2* mutants

4) It is also not clear how it affects microtubule cytoskeleton. The primary effect could be microtubule cytoskeleton as well.

We thank this reviewer for this suggestion. We have discussed this possibility in the discussion.

In the high-ordered branches, loss of *chdp-1* reduced not only the cortical actin assembly, but also the microtubule assembly. Two models can explain the decreased microtubule cytoskeleton. (1) CHDP-1 directly acts as a microtubule assembly factor. (2) The effect of CHDP-1 on microtubule assembly is secondary of its function in cortical actin assembly. Actin cytoskeleton often interacts with microtubule cytoskeleton [1,47]. Numerous proteins have been reported to mediate structural interactions between microtubules and actin, such as coronin, and Drebrin-EB3 [48,49]. Disrupting of actin cytoskeleton can lead to microtubule assembly defects [50]. Thus, it is conceivable to hypothesize that the cortical actin in the high-ordered branches can serve as a platform to promote

microtubule assembly and stabilization. Future studies are needed to tell which model is correct.

5) Related to this question: The ectopic 2o dendrites could be due to the over stabilization of microtubules. Or is it due to the instability of microtubules. Treating the mutant with colchicine or taxol might shed some light.

We thank this reviewer for this suggestion. We have followed a protocol reported by Leonie Kirszenblat et al. (2012, MBoC) and treated the *chdp-1* mutants with colchicine or taxol, and did not see any enhanced or suppressed dendrite morphogenesis defects (please see Data 5).

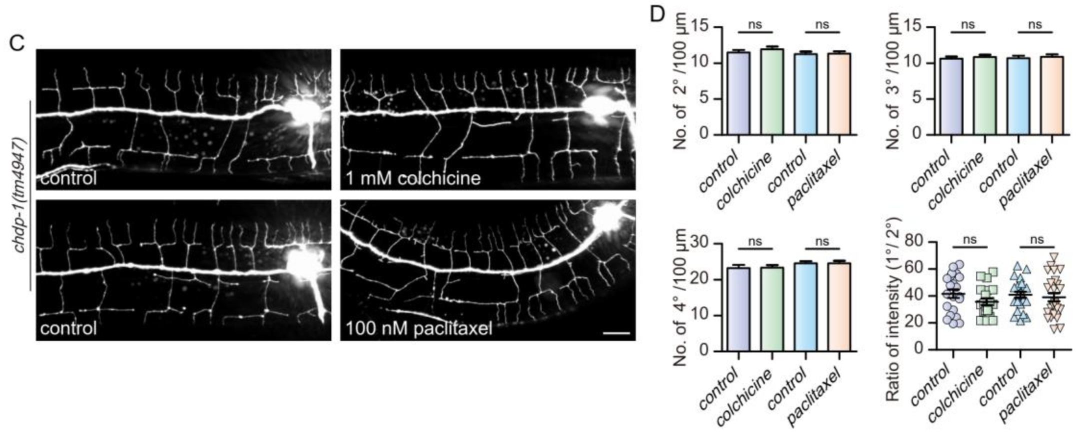

Data 5: PVD morphologies of *chdp-1* mutant animals treated with MT drugs

6) Thickening of the primary dendrites could be a sign of abnormal microtubule cytoskeleton. Although according to the Supple Figure 6, authors conclude that EBP-2 tracks in primary dendrites are unaffected, I recommend to show a better kymograph from the mutant. It looks like there is some movement during imaging. I also suggest to look at the growth duration and length of these tracks in wild type and mutant. Also check the pause frequency. It might tell whether there is any effect on growth or depolymerization.

We thank this reviewer for this suggestion. We have showed a better kymograph for the mutant. We also have quantified the growth duration, length of the tracks, and the pause frequency. We did not see any difference between the WT and *chdp-1* mutants (please see S11 Fig).

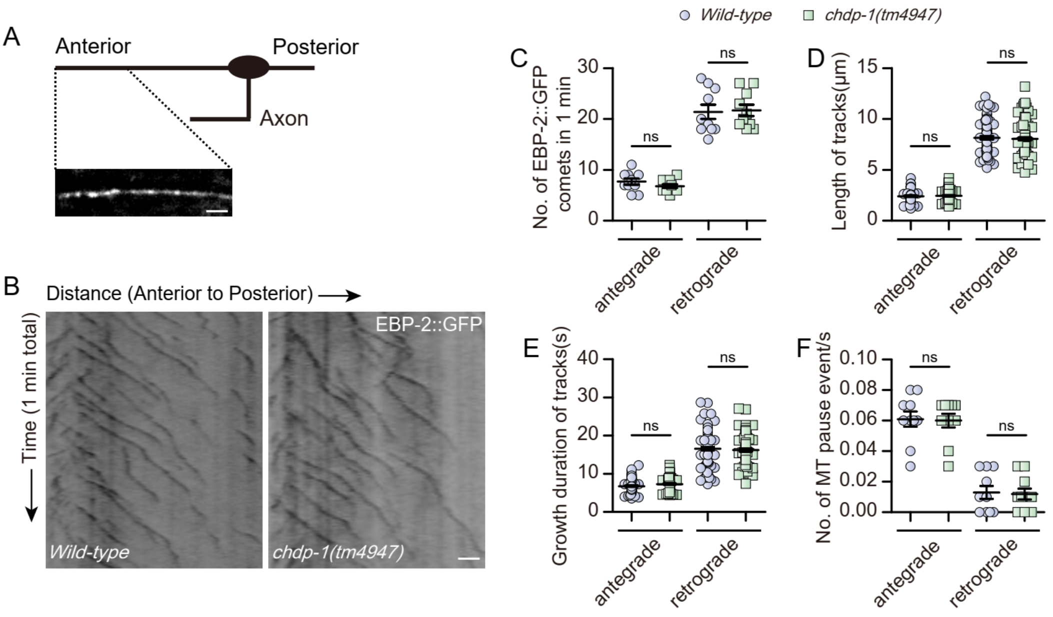

Part of S11 Fig: MT assembly in the 1° dendrites is not affected by knock-out of *chdp-1*

7) Author may discuss the possibility that CHDP-1 can affect microtubule cytoskeleton directly in an unknown mechanism. Since it is not tested yet whether it associates with microtubule or not, this possibility always remains. Although I am not asking to do more experiments on this.

We thank this reviewer for this suggestion. We have generated a strain to simultaneously examine the subcellular localization of CHDP-1 and TBA-1. It seems that some MTs are co-localized with CHDP-1 near the cell cortex (Data 2). Thus, it is possible that CHDP-1 directly associates with MTs and regulates MT assembly. We also discussed about this possibility in the discussion.

In the high-ordered branches, loss of *chdp-1* reduced not only the cortical actin assembly, but also the microtubule assembly. Two models can explain the decreased microtubule cytoskeleton. (1) CHDP-1 directly acts as a microtubule assembly factor. (2) The effect of CHDP-1 on microtubule assembly is secondary of its function in cortical actin assembly. Actin cytoskeleton often interacts with microtubule cytoskeleton [1,47]. Numerous proteins have been reported to mediate structural

interactions between microtubules and actin, such as coronin, and Drebrin-EB3 [48,49]. Disrupting of actin cytoskeleton can lead to microtubule assembly defects [50]. Thus, it is conceivable to hypothesize that the cortical actin in the high-ordered branches can serve as a platform to promote microtubule assembly and stabilization. Future studies are needed to tell which model is correct.

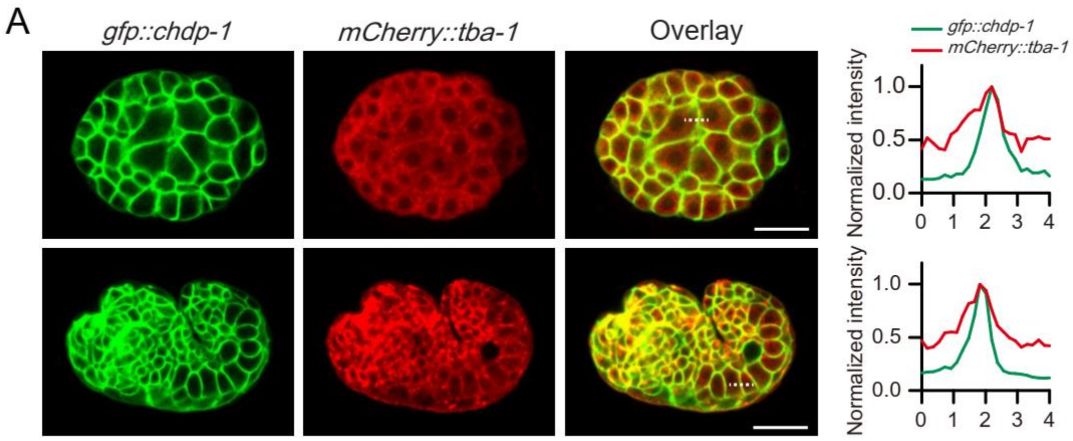

Data 2: Co-labeling of CHDP-1 and TBA-1 in embryos

8) Did they check the synaptic vesicles in the mutant dendrites? RAB-3 reporters?

We thank this reviewer for this suggestion. We have generated and checked the RAB-3 reporter for the synaptic vesicles in the mutant dendrites. We did not see any mis-localization of RAB-3 in the dendrites, suggesting that the MT polarity in the primary dendrites is not affected by *chdp-1* knock-out. Please see S11 Fig.

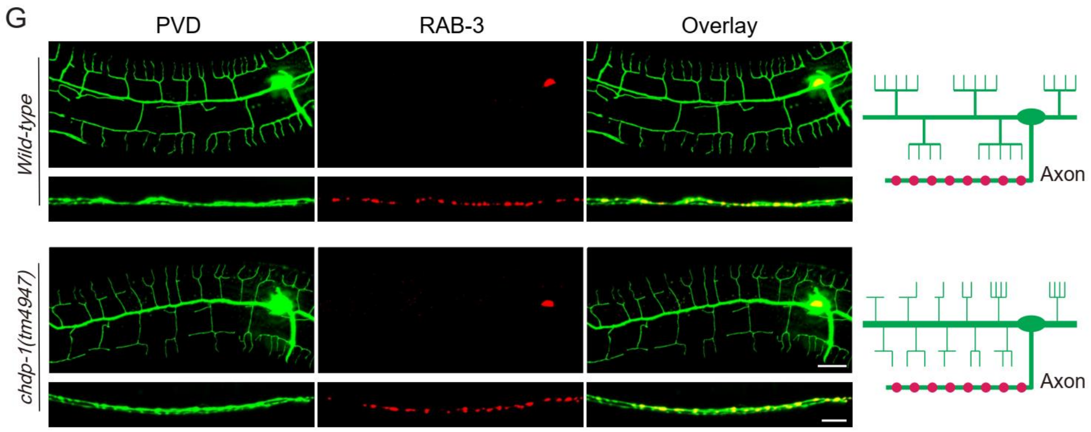

Part of S11 Fig: Synaptic vesicles are not mis-localized into dendrites in *chdp-1* mutants

9) The connection with SAX-1 is also not clear. Activity or amount of SAX-1 might be upregulated in chdp-1 mutant? Is there any SAX-1 reporter available to test? If it is possible, it can be addressed.

We thank this reviewer for this suggestion. Since loss of sax-1 can suppress the dendrite development defects of *chdp-1* mutants, it seems to us that *sax-1* acts either in parallel or downstream of *chdp-1*. We do agree with this reviewer that it might be interesting to see whether the activity or amount of SAX-1 might be upregulated in the *chdp-1* mutants. It is difficult to examine the activity of this kinase. So, we made a SAX-1::GFP reporter using CRISPR/Cas9 genome editing. We found that the protein level of endogenous SAX-1 is similar in WT and *chdp-1* mutants. Please see Data 6.

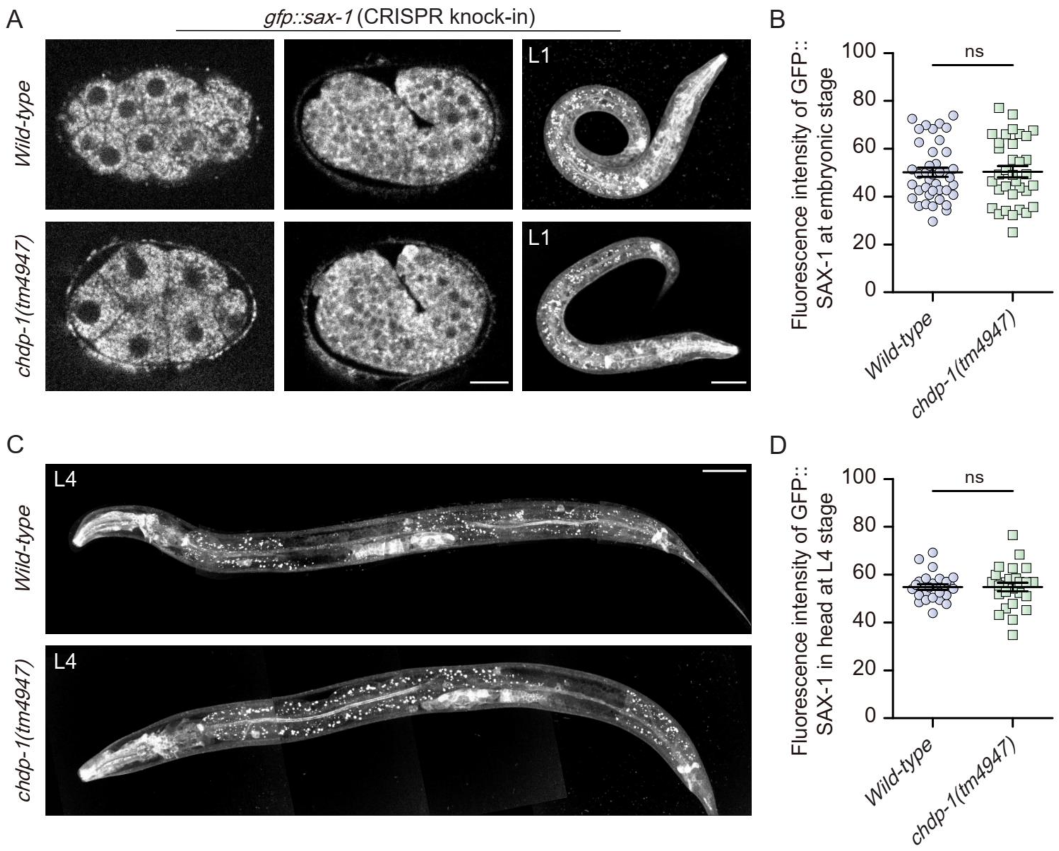

Data 6. Expression levels of endogenous SAX-1 in WT and *chdp-1* mutant animals
